# Rhoptry neck protein 4 plays important roles during *Plasmodium* sporozoite infection of the mammalian liver

**DOI:** 10.1128/msphere.00587-22

**Published:** 2023-06-05

**Authors:** Minami Baba, Mamoru Nozaki, Mayumi Tachibana, Takafumi Tsuboi, Motomi Torii, Tomoko Ishino

**Affiliations:** 1 Division of Molecular Parasitology, Proteo-Science Center, Ehime University, Toon, Ehime, Japan; 2 Department of Protozoology, Institute of Tropical Medicine (NEKKEN), Nagasaki University, Sakamoto, Nagasaki, Japan; 3 Division of Malaria Research, Proteo-Science Center, Ehime University, Matsuyama, Ehime, Japan; 4 Department of Parasitology and Tropical Medicine, Graduate School of Medical and Dental Sciences, Tokyo Medical and Dental University (TMDU), Yushima, Bunkyo-ku, Tokyo, Japan; Weill Cornell Medicine, New York, New York, USA

**Keywords:** malaria, *Plasmodium*, sporozoite, rhoptry, invasion

## Abstract

During invasion, *Plasmodium* parasites secrete proteins from rhoptry and microneme apical end organelles, which have crucial roles in attaching to and invading target cells. A sporozoite stage-specific gene silencing system revealed that rhoptry neck protein 2 (RON2), RON4, and RON5 are important for sporozoite invasion of mosquito salivary glands. Here, we further investigated the roles of RON4 during sporozoite infection of the liver *in vivo*. Following intravenous inoculation of RON4-knockdown sporozoites into mice, we demonstrated that sporozoite RON4 has multiple functions during sporozoite traversal of sinusoidal cells and infection of hepatocytes. *In vitro* infection experiments using a hepatoma cell line revealed that secreted RON4 is involved in sporozoite adhesion to hepatocytes and has an important role in the early steps of hepatocyte infection. In addition, *in vitro* motility assays indicated that RON4 is required for sporozoite attachment to the substrate and the onset of migration. These findings indicate that RON4 is crucial for sporozoite migration toward and invasion of hepatocytes via attachment ability and motility.

**IMPORTANCE:**

Malarial parasite transmission to mammals is established when sporozoites are inoculated by mosquitoes and migrate through the bloodstream to infect hepatocytes. Many aspects of the molecular mechanisms underpinning migration and cellular invasion remain largely unelucidated. By applying a sporozoite stage-specific gene silencing system in the rodent malarial parasite, *Plasmodium berghei*, we demonstrated that rhoptry neck protein 4 (RON4) is crucial for sporozoite infection of the liver *in vivo*. Combined with *in vitro* investigations, it was revealed that RON4 functions during a crossing of the sinusoidal cell layer and invading hepatocytes, at an early stage of liver infection, by mediating the sporozoite capacity for adhesion and the onset of motility. Since RON4 is also expressed in *Plasmodium* merozoites and *Toxoplasma* tachyzoites, our findings contribute to understanding the conserved invasion mechanisms of *Apicomplexa* parasites.

## INTRODUCTION

*Plasmodium* parasites are in the phylum *Apicomplexa* and are the causative agents of malaria, a devastating infectious disease transmitted via mosquitoes. Approximately 600,000 people worldwide die from malaria each year (1). The life cycle of the *Plasmodium* parasite includes forms infectious for intracellular development, and one of which, the merozoite, mediates invasion and subsequent proliferation within erythrocytes. This erythrocytic infection cycle causes clinical symptoms such as anemia and fever. The sporozoite is the second such infective form and is transmitted from a vector mosquito to a mammalian host and ultimately invades hepatocytes.

To make a successful infection of host cells, apicomplexan parasites utilize a common structural organization at their anterior pole, called the apical complex (2, 3), which includes secretory organelles termed rhoptries and micronemes. Some secretory proteins stored in rhoptries are conserved among infectious forms of apicomplexan parasites, such as *Plasmodium* merozoites and *Toxoplasma* tachyzoites, and have roles during target cell invasion (4). For example, during merozoite invasion of erythrocytes, rhoptry neck protein (RON) 2, RON4, and RON5 are secreted to the host cell membrane to form the RON complex, which interacts with AMA1 on the parasite membrane to create a moving junction (5 - 9). The importance of the interaction between RON2 and AMA1 for merozoite invasion of erythrocytes has been shown using inhibitory antibodies and peptides (10 - 13). The inability to target gene disruption of *ron2*, *ron4*, *ron5*, or *ama1* supports that these genes are crucial for merozoite invasion (14).

Sporozoites are formed in oocysts located between the epithelium and basal lamina of the mosquito midgut. After release into the hemolymph, sporozoites adhere to and invade salivary glands prior to transmission to mammalian hosts. Sporozoites are deposited into the skin during mosquito probing, and then actively migrate through the skin to enter blood vessels (15, 16). During passage through the liver, sporozoites recognize and traverse the sinusoidal cell layer to reach target hepatocytes (17, 18). Thus, sporozoites have a mechanism to migrate toward hepatocytes through different types of cells, in addition to infecting and differentiating within hepatocytes.

Both sporozoites and merozoites contain rhoptries at the apical end, in which proteins such as RON2, RON4, and RON5 are expressed (19). To elucidate the roles of RON2, RON4, and RON5 in sporozoites, we developed a sporozoite stage-specific knockdown system using the promoter swapping method (20). Repression of RON2, RON4, or RON5 expression in sporozoites demonstrated that the proteins are crucial for sporozoite invasion of salivary glands (20, 21). The importance of RON2 and RON4 in sporozoite invasion of salivary glands was confirmed by a conditional knockout system based on DiCre recombinase (22). These results raised the question of whether conserved rhoptry proteins are also required for sporozoite transmission from mosquitoes to mammals, specifically, migration toward and infection of hepatocytes.

Following the sporozoite invasion of hepatocytes, the parasites differentiate into liver stages (LS) and mature to form several thousand liver merozoites within a parasitophorous vacuole membrane (PVM). Risco-Castillo et al. reported that RON2 and RON4 are discharged during sporozoite invasion of hepatocytes (23), suggesting their importance for hepatocyte infection. By applying a promoter swapping strategy, we demonstrated that RON2 is involved in infection of the liver *in vivo*, as well as in infection of hepatocytes *in vitro* (20). In the case of RON4, only *in vitro* experiments were performed, using a conditional mutant parasite prepared by the FLP-FRT (flippase/flippase recombination target site) recombination system or DiCre recombinase and suggested that RON4 is involved in the infection of hepatocytes (24). However, the physiological roles of RON4 in sporozoite transmission to the liver remain unclear *in vivo*, including a potential role in migration toward hepatocytes.

In this study, the detailed functions of RON4 in sporozoite transmission to the liver were analyzed *in vivo* and *in vitro* using RON4 conditional knockdown (RON4-cKD) sporozoites, in which RON4 protein expression was suppressed to undetectable levels. The results revealed that RON4 has multiple roles during the sporozoite crossing of sinusoidal cells to arrive at hepatocytes, the adhesion of hepatocytes, and the invasion of hepatocytes, all of which are essential steps for sporozoite transmission from the mosquito to the mammalian liver.

## MATERIALS AND METHODS

### Parasites and mosquitoes

In this study, a transgenic *Plasmodium berghei (Pb*) ANKA parasite line (*Pb*ANKA-GFP) was used which constitutively expresses green fluorescent protein (GFP) under the control of the *elongation factor 1A* (*ef1α*) promoter without a drug resistance gene (25), kindly given by Dr. Janse. Transgenic *Pb* parasites in which the native *ron4* promoter region was replaced by a promoter region for *pb merozoite surface protein 9 (pbmsp9*) or native *pbron4*, with a drug resistance cassette (named RON4-cKD and RON4-cont, respectively) were generated as described (21).

Cryopreserved *Pb*ANKA*-*infected erythrocytes were intraperitoneally injected into female ICR mice (4, 6 weeks old; CLEA Japan, Tokyo, Japan) to obtain blood-stage parasites. *Anopheles stephensi* SDA 500 strain mosquitoes were maintained on a 5% sucrose solution at 25°C. After feeding on *Pb*-infected ICR mice, fully engorged mosquitoes were selected and kept at 20°C under a 12-hour light/12-hour dark cycle until dissection. All animal experimental protocols were approved by the Institutional Animal Care and Use Committee of Ehime University and Tokyo Medical and Dental Univerity, and the experiments were conducted according to the Ethical Guidelines for Animal Experiments of Ehime University and Tokyo Medical and Dental University.

### Generation of mCherry-tagged RON4 expressing transgenic parasites

To generate transgenic parasites expressing mCherry-tagged RON4 at its C-terminus, the native *ron4* locus in the *Pb*ANKA-GFP genome was replaced by a single crossover homologous recombination with an expression cassette of the C-terminus of the *ron4* coding region fused with an mCherry tag, similar to the generation of RON4-c-Myc expressing parasites (19). A schematic representation of the transgenic vector construction is shown in Fig. S1A. The c-Myc tag sequence was replaced by the mCherry tag sequence in the pL0033 plasmid (BEI Resources, Manassas, VA, USA), and then 1,626 base pairs corresponding to the RON4 C-terminus region, containing an XbaI site in the middle introduced by site-directed mutagenesis as described (19), was inserted at SacII and BamHI sites just upstream of the mCherry-tag coding region. Electroporation of 10–15 µg linearized DNA by XbaI digestion into a schizont-enriched *Pb*ANKA-GFP line and selection of transgenic parasites was performed as described (26). DNA integration occurs at the target locus in the *Pb*ANKA-GFP genome by single crossover homologous recombination as illustrated in Fig. S1A. Correct DNA integration into the target locus was confirmed by PCR genotyping, and transgenic parasites were cloned by limiting dilution.

### Western blotting analysis

Sporozoites collected from the midguts of infected mosquitoes at days 18 to 21 post-feeding were purified by density gradient centrifugation using a 17% Accudenz solution (Accurate Chemical & Scientific Corporation, NY, USA) (27). Sporozoite pellets were resuspended and sonicated in a sample buffer solution for SDS-PAGE (nacalai tesque, Kyoto, Japan) containing 5% 2-mercaptoethanol. Sporozoites collected from salivary glands were directly resuspended in a sample buffer for SDS-PAGE. Proteins were separated by SDS-PAGE using 12.5% acrylamide gels (ATTO, Tokyo, Japan) and electroblotted onto polyvinylidene difluoride (PVDF) membranes. The PVDF membranes were blocked with Blocking One (nacalai tesque) for 1 hour at room temperature and then incubated overnight at 4°C with primary antibodies (1:500 anti-mCherry rabbit polyclonal antibodies [abcam, Cambridge, UK]; specifically, 1:1,000 anti-RON4 [21] or 1:20,000 anti-Heat Shock Protein 70 [HSP70] rabbit polyclonal antibodies [20]) diluted in phosphate-buffered saline (PBS) containing 1% skim milk (Megmilk Snow Brand, Tokyo, Japan). After washing with PBS containing 0.01% Tween-20 (PBST), the membranes were incubated with anti-rabbit IgG secondary antibodies conjugated to horseradish peroxidase (HRP; 1:30,000; Biosource, Camarillo, CA, USA) for 30 minutes at room temperature. Chemiluminescence detection was performed by adding Immobilon Western Chemiluminescent HRP Substrate (Merck Millipore, Darmstadt, Germany), and the signal was detected using an ImageQuant LAS 4010 (GE Healthcare, Chalfont St Giles, UK).

### Confocal microscopy observation of sporozoites and liver-stage parasites

To investigate the expression pattern of RON4 in LS parasites, RON4-mCherry sporozoites were collected from salivary glands at days 25 to 28 post-feeding. Then, 80,000– 150,000 sporozoites were inoculated onto confluently seeded HepG2 cells, a human hepatoma cell line, in an eight-well chamber slide; then incubated in culture medium (RPMI 1640 medium [FUJIFILM Wako Pure Chemical, Osaka, Japan] containing 10% fetal calf serum [FCS], 100 IU/mL penicillin, and 100 µg/mL streptomycin [FUJIFILM Wako]) at 37°C in the presence of 5% CO_2_ for 24, 48, or 72 hours prior to observation. The RON4-mCherry sporozoites were also sowed directly onto glass bottom dishes just before observation. To detect nuclei, samples were stained with Hoechst 33342 (Sigma-Aldrich, St. Louis, USA) and observed using a confocal scanning laser microscope (LSM710; Carl Zeiss MicroImaging, Oberkochen, Germany).

### Real-time reverse transcription (RT)-PCR

To confirm the transcript levels of *ron4* in LS parasites, real-time RT-PCR was carried out using specific primers as shown in Table S1. Five or ten thousand sporozoites were collected from the salivary glands of *Pb*ANKA-GFP-infected mosquitoes at days 21 to 24 post-feeding and were injected intravenously into female C57BL/6 mice (CLEA Japan). For total RNA extraction, 24, 40, or 48 hours post-inoculation the livers were perfused and homogenized in 5 mL of Trizol (Thermo Fisher Scientific, San Jose, CA, USA) using a polytron homogenizer (Kinematica AG, Luzern, Switzerland). As a control, the infected mosquito salivary glands at day 17 post-feeding were collected in RNAlater (Thermo Fisher Scientific) and stored at 4°C until RNA isolation. Total RNA was extracted from infected mosquito salivary glands using an RNeasy Micro Kit (Qiagen GmbH, Hilden, Germany). After DNase treatment, cDNA was synthesized using a PrimeScript RT reagent Kit (Perfect Real Time; Takara Bio, Otsu, Japan). Real-time PCR was performed using a TaKaRa PCR Thermal Cycler Dice (Takara Bio) with an SYBR Premix Ex Taq (Takara Bio) and *ron4*-specific primers (Table S1; *Pb*RON4 RT-F and R). Relative gene expressions were normalized by *ef1α* (PBANKA_1133300; Table S1; *Pb*EF1a RT-F and R) mRNA levels and were compared using the comparative Ct method (19, 20, 28).

### Indirect immunofluorescence assay (IFA)

Sporozoites were collected from salivary glands at days 19 to 22 post-feeding and seeded on eight-well multi-well slides, then air-dried and fixed with cold acetone for 3 minutes. The slides were blocked with Blocking One Histo (nacalai tesque) at 37°C for 30 minutes and incubated with primary antibodies (1:2,000 for anti-RON4 rabbit polyclonal antibodies, and anti-circumsporozoite protein (CSP) mouse monoclonal antibodies; MRA-100, obtained through BEI Resources, NIAID, NIH) in PBST containing 5% Blocking One Histo at 37°C for 2 hours, followed by Alexa Fluor 488–conjugated goat anti-rabbit IgG and Alexa Fluor 546–conjugated goat anti-mouse IgG (1:500, Thermo Fisher Scientific) at 37°C for 30 minutes. Nuclei were stained with 2 µg/mL of 4′,6-diamidino-2-phenylindole (DAPI). The samples were mounted in ProLong Gold antifade reagent (Thermo Fisher Scientific) and observed with an inverted fluorescence microscope (Axio Observer Z1; Carl Zeiss, Oberkochen, Germany).

### *In vivo* liver infectivity

*In vivo* liver infectivity was performed as described (20). Briefly, 30,000 sporozoites were collected from salivary glands at days 20 to 23 post-feeding and injected intravenously into a female C57BL/6 mouse (CLEA Japan). One or 42 hours post-inoculation, the livers were perfused and homogenized in 5 mL of Trizol for total RNA extraction, followed by real-time RT-PCR analysis as described above (see the section “Real-time reverse transcription (RT)-PCR”). Experiments were performed using RON4-cont, independent knockdown clones: RON4-cKD cl1 and cl2, with 5 or 10 mice for each parasite line. The difference in the relative *Pb*18S rRNA levels between RON4-cont and RON4-cKD, after normalization using murine *gapdh* mRNA amounts, was analyzed by the Mann–Whitney *U* test. The primers used in these experiments are listed in Table S1 (*Pb*18S rRNA RT-F and R, *Mm*GAPDH RT-F and R).

### Determination of blood-stage parasitemia after sporozoite inoculation

Sporozoites were collected from salivary glands at days 19 to 21 post-feeding and injected intravenously into female C57BL/6 mice (CLEA Japan). The parasitemias of infected mice were monitored by daily blood smears from days 3 to 7 post-infection. Blood smears were stained in Giemsa solution, and infected erythrocytes were counted using a light microscope.

### *In vitro* sporozoite cell traversal assay

Cell traversal ability was assayed by the number of wounded cells 1 hour after sporozoite inoculation as described (29). Briefly, 10,000 hemocoel sporozoites were incubated for 1 hour on confluent 3T3-Swiss albino cells in an eight-well chamber slide, with 2 mg/mL fluorescein-conjugated dextran (molecular weight 10,000, lysine fixable; Thermo Fisher Scientific) in RPMI 1640 medium containing 10% FCS. Cells were washed with PBS and fixed with 10% formalin. Fluorescence-labeled cells were counted under a fluorescence microscope (Axio Observer Z1). Experiments were repeated five times with more than three wells for each parasite line.

### *In vitro* sporozoite invasion analyses

The numbers of extracellular and intracellular sporozoites of HepG2 cells were examined by an invasion assay using two kinds of anti-CSP antibodies (30, 31). To distinguish between extracellular and intracellular sporozoites, 10,000 sporozoites from RON4-cont, RON4-cKD cl1, and RON4-cKD cl2 lines were collected from salivary glands at days 16 to 23 post-feeding and inoculated onto HepG2 with culture medium in eight-well chamber slides. After adding sporozoites, slides were centrifuged at 500 × *g* for 5 minutes at room temperature and incubated for 1 hour at 37°C with 5% CO_2_. Cells were fixed with 10% formalin. The slides were blocked with 10% FCS at 37°C for 30 minutes and extracellular sporozoites were labeled with anti-rabbit CSP-repeat region antibodies, followed by Alexa Fluor 546–conjugated goat anti-rabbit IgG antibodies (1:500). The cells were then permeabilized with 0.1% Triton X-100 and re-labeled using anti-CSP mouse monoclonal antibodies followed by Alexa Fluor 488–conjugated goat anti-mouse IgG antibodies (1:500). The numbers of extracellular and intracellular sporozoites were examined by quantification of double- or single-stained sporozoites under a fluorescence microscope (Axio Observer Z1). Experiments were repeated four times with more than three wells for each parasite line.

For infection assays, five thousand sporozoites from the RON4-cont, RON4-cKD cl1, and RON4-cKD cl2 lines were collected from salivary glands at days 16 to 23 post-feeding and were inoculated onto HepG2 cells as described above and incubated for 6 or 48 hours at 37°C with 5% CO_2_. Cells were fixed with 10% formalin and permeabilized by 0.1% Triton X-100. The slides were blocked with 10% FCS at 37°C for 30 minutes and incubated with primary antibodies (1:500 for anti-upregulated in infective sporozoites 4 (UIS4) rabbit polyclonal antibodies, and anti-CSP mouse monoclonal antibodies) in PBS containing 10% FCS at 37°C for 2 hours, followed by Alexa Fluor 546–conjugated goat anti-rabbit IgG and Alexa Fluor 488–conjugated goat anti-mouse IgG at 37°C for 30 minutes. Nuclei were stained with 2 µg/mL of DAPI. The number of infected sporozoites (6 hours) and LS parasites (48 hours) were counted. Experiments were repeated five times with more than three wells for each parasite line.

### *In vitro* sporozoite attachment and motility assay

RON4-cont, RON4-cKD cl1, and RON4-cKD cl2 sporozoites were collected from salivary glands at days 19 to 26 post-feeding. For attachment and motility assays on glass slides, sporozoites in RPMI 1640 medium containing 10% FCS were placed in a glass-bottom dish. For gliding assays in Matrigel, sporozoites in RPMI 1640 containing 20% FCS were mixed with an equal volume of Matrigel (Corning, NY, USA) and placed on glass bottom dishes. Sporozoite movement was detected by GFP fluorescence using an Axio Observer Z1 and recorded with an AxioCam MRm charge-coupled device camera (Carl Zeiss) every 2 seconds for up to 150 frames (5 minutes). Sporozoites on glass slides were classified manually according to their attachment patterns as follows: full-length-attached, partial-attached, or non-attached. Full-length-attached sporozoites were further categorized depending on their motility. Experiments were repeated five times with at least 80 sporozoites per parasite line. The effect of RON4 repression on sporozoite attachment and motility was evaluated by comparison with RON4-cont sporozoites. In Matrigel, sporozoites were categorized as moving in a circular mode (circulating), meandering, or non-motile, according to the work of Nozaki et al. (21). Experiments were repeated four times with at least 40 sporozoites for each line. The velocity of gliding sporozoites was calculated using the MTrack2 plugin in Fiji software (https://imagej.net/plugins/mtrack2).

### RON4 discharge during sporozoite migration

To investigate the discharge of RON4 protein from sporozoites during migration, RON4-cont sporozoites collected from salivary glands on day 21 post-feeding were suspended in RPMI 1640 containing 10% FCS and transferred to eight-well chamber slides. After adding sporozoites, slides were centrifuged at 500×*g* for 5 minutes at room temperature and incubated for 1 hour at 37°C with 5% CO_2_. Slides were washed with PBS and fixed with 2% paraformaldehyde. The slides were blocked with Blocking One Histo at 37°C for 30 minutes and incubated with primary antibodies (1:2,000 for anti-RON4 rabbit polyclonal antibodies, and anti-CSP mouse monoclonal antibodies) in PBS containing 5% Blocking One Histo at 37°C for 2 hours, followed by Alexa Fluor 488–conjugated goat anti-rabbit IgG (1:500) and Alexa Fluor 546–conjugated goat anti-mouse IgG (1:500) at 37°C for 30 minutes. Nuclei were stained with Hoechst 33342 (1:500).

### Antibody inhibition against sporozoite invasion of hepatocytes

To investigate the effects of anti-RON4 antibodies during sporozoite invasion of hepatocytes, 20,000 or 30,000 *Pb*ANKA-GFP sporozoites were collected from salivary glands at days 20 to 28 post-feeding and inoculated onto HepG2 cells with medium containing anti-RON4 or anti-glutathione *S*-transferase (GST) rabbit IgG at 62.5 µg/mL, 125 µg/mL, 250 µg/mL, 500 µg/mL, or 1 mg/mL and incubated for 1.5 hours in culture medium in an eight-well chamber slide. For IgG purification, antiserum against RON4 or GST was applied to protein G columns (GE Healthcare, NJ, USA) according to the manufacturer’s instructions. As a negative control, PBS was added instead of IgG. The cells were washed with the culture medium three times and further incubated for 4.5 hours with the culture medium at 37°C with 5% CO_2_. After a total of 6 hours of incubation, cells were washed with PBS and fixed with 10% formalin and permeabilized by 0.1% Triton X-100. The parasites were detected by IFA using antibodies against UIS4 and CSP as described above. The number of infected parasites identified as PVM-positive was counted. Experiments were repeated four times with three wells for Fig. 6 and twice with two wells for the antibody dose dependency in Fig. S9.

### Statistical analysis

All statistical analyses were performed using Prism 9 software (GraphPad, version 9. 3. 1 [350]). All data were checked for normality and homogeneity of variance to analyze accordingly using parametric or non-parametric test. Mann–Whitney *U* test was used for comparisons between the two groups. One-way analysis of variance (ANOVA), Brown–Forsythe test, or Kruskal–Wallis test were performed to compare more than three groups. Dunnett’s multiple comparison test was used as the *post hoc* for parametric tests, and Dunn’s *post hoc* test was used as the *post hoc* for non-parametric tests. Statistical significance was set at *P* < 0.05.

## RESULTS

### RON4 expression pattern in sporozoites and liver-stage parasites

**Fig 1:**
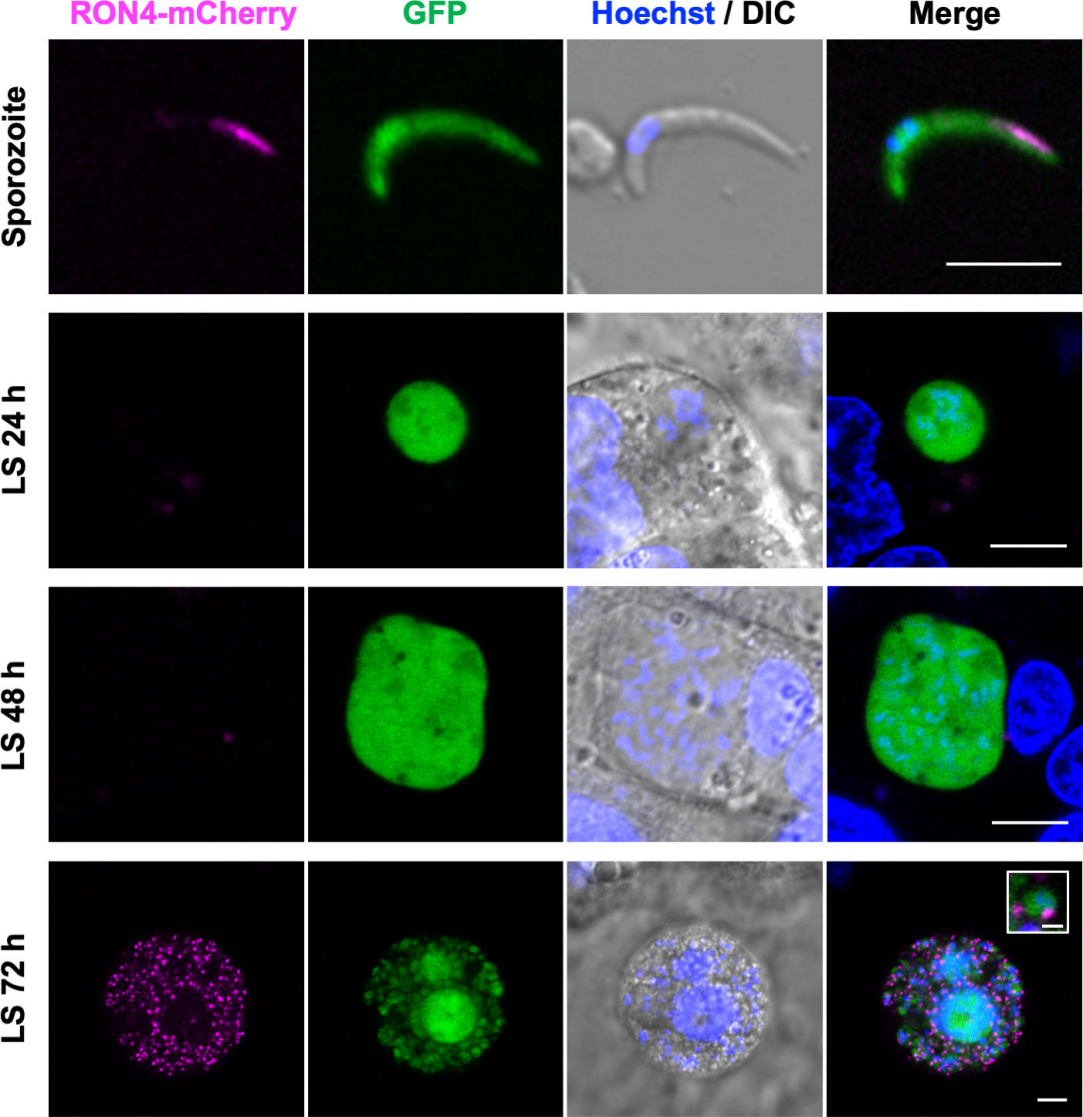
RON4 expression pattern in sporozoites and liver-stage (LS) parasites. Expression of RON4-mCherry in sporozoites and liver-stage parasites. RON4-mCherry sporozoites were collected from salivary glands at days 25 to 28 post-feeding. The sporozoites were inoculated on HepG2 cells in culture medium in glass bottom dishes and then incubated for 24 hours (LS 24 h), 48 hours (LS 48 h), and 72 hours (LS 72 h). Sporozoites were also inoculated on a glass bottom dish without cells (sporozoites). Live parasites were detected by cytosol GFP signal (green). RON4 was detected by an mCherry signal (magenta). Nuclei were stained with Hoechst 33342 (blue). Scale bars, 5 µm. The inserted image demonstrates an enlarged liver merozoite (scale bar, 1 µm).

RON4 is expressed and localized to rhoptries in both merozoites and sporozoites of *Plasmodium berghei* (19). To investigate RON4 contribution to parasite transmission from mosquitoes to mammals, the RON4 expression pattern was examined in the LS by generating a transgenic *P. berghei* ANKA parasite line which expresses mCherry-tagged RON4 instead of the RON4 native coding sequence (RON4-mCherry; see the section “Materials and Methods” and Fig. S1A). The successful expression of mCherry-tagged RON4 in oocyst-derived sporozoites was demonstrated by western blot using anti-RON4 and anti-mCherry antibody staining (Fig. S1B). Two bands corresponding to full-length RON4 (about 100 kDa) and a processed form (about 60 kDa) were observed on western blots, both of which were shifted due to the successful addition of an mCherry-tag at the C-terminus. Inoculating sporozoites collected from mosquito salivary glands to C57BL/6 mice demonstrated that RON4-mCherry-expressing sporozoites could normally infect and proliferate within hepatocytes (Fig. S1C). By confocal fluorescence microscopy, RON4-mCherry signals were confirmed to localize at the apical end of sporozoites residing in salivary glands, consistent with a rhoptry localization (Fig. 1, top row) (21). To examine RON4-mCherry expression in LS parasites, RON4-mCherry sporozoites collected from salivary glands were inoculated onto the human hepatoma cell line, HepG2. At 24 and 48 hours post-inoculation, RON4-mCherry signals were undetectable in LS parasites (Fig. 1, second and third rows). At 72-hour post-inoculation, RON4-mCherry signals were detected as dots corresponding to the tip of mature liver merozoites (Fig. 1, bottom row).

Next, the *ron4* mRNA expression pattern during LS parasite development was measured *in vivo*, by intravenous inoculation of *Pb*ANKA-GFP sporozoites into C57BL/6 mice. The relative *ron4* mRNA amounts in sporozoites and in LS parasites were examined by real-time RT-PCR at 24, 40, and 48 hours post-sporozoite inoculation and normalized by *Plasmodium ef1α* mRNA expression. While *ron4* mRNA transcription was clearly detected in sporozoites collected from salivary glands, as reported (19), *ron4* mRNA was undetectable in LS parasites at 24 and 40 hours after sporozoite inoculation (Fig. S2; LS 24 h and LS 40 h). *Ron4* mRNA became detectable at the late LS, 48 hours after sporozoite inoculation (Fig. S2; LS 48 h), a timepoint at which merozomes, infected hepatocytes filled with matured merozoites, begin sequestration into blood vessels to release merozoites (32). These data demonstrated that *ron4* transcription is upregulated significantly on hepatic-merozoite maturation, which is consistent with protein detection results (Fig. 1). Since *ron4* transcription in the LS is repressed until hepatic merozoites are mature, RON4 is unlikely to be required for parasite proliferation in hepatocytes. Therefore, we further investigated the role of RON4 in sporozoites in terms of mosquito-to-mammal transmission.

### RON4 is required for sporozoite infection of the liver

**Fig 2:**
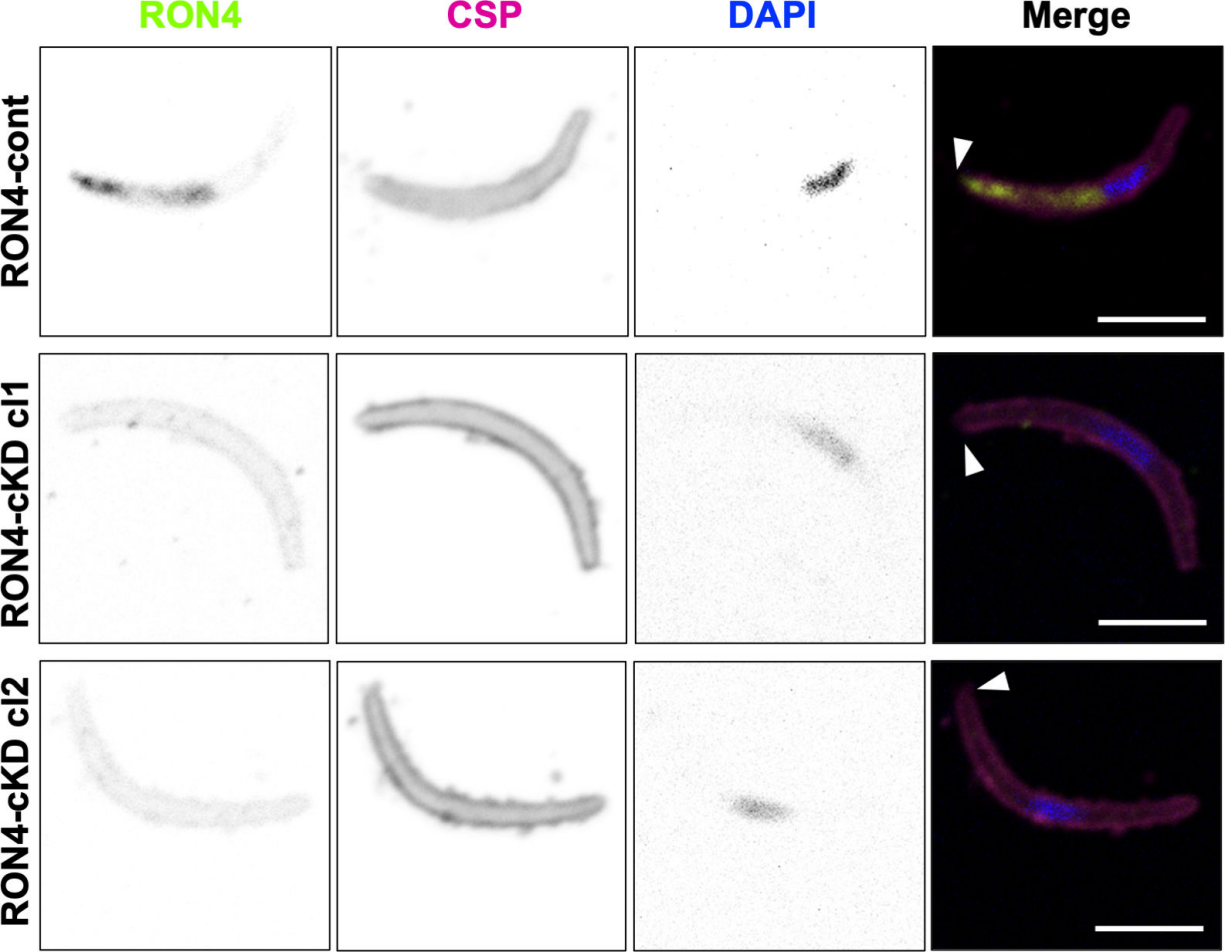
RON4 signal is undetectable in sporozoite stage-specific ron4 knockdown parasites collected from salivary glands. Indirect immunofluorescence analysis of RON4. Sporozoites collected from salivary glands were stained with anti-RON4 and anti-CSP antibodies with nuclei visualized with 4’,6-diamidino-2-indole (DAPI). The individual signals are shown in black and white. Merged images in the right columns contain RON4 (shown in green), CSP (purple), and DAPI (blue). In RON4-cont sporozoites, the specific signals corresponding to RON4 were detected at the apical end; however, these signals were undetectable at the apical end (arrowheads) of RON4-cKD sporozoites. Bar, 5 µm.

To investigate RON4 roles during sporozoite infection of the liver, sporozoite stage-specific *ron4* knockdown (RON4-cKD) *P. berghei* ANKA parasites were used, which were previously generated by replacing the native *ron4* promoter with a merozoite surface protein 9 promoter (21). RON4-cKD oocyst-derived sporozoites repress *ron4* transcription to levels approximately 70-fold less than *Pb*ANKA-GFP and result in a roughly 30-fold lower ability to invade salivary glands (21). To determine whether the small number of RON4-cKD sporozoites that successfully invade salivary glands is due to the leaky expression of RON4, an IFA was performed on RON4-cKD sporozoites collected from salivary glands. As shown in Fig. 2, the specific signal corresponding to RON4 detected at the apical end of RON4-cont sporozoites could not be detected in RON4-cKD sporozoites. The decrease in RON4 protein amount was confirmed by Western blotting (Fig. S3). Therefore, we used RON4-cKD sporozoites collected from salivary glands for further experiments to investigate RON4 involvement in sporozoite infection of mammalian livers.

**Fig 3:**
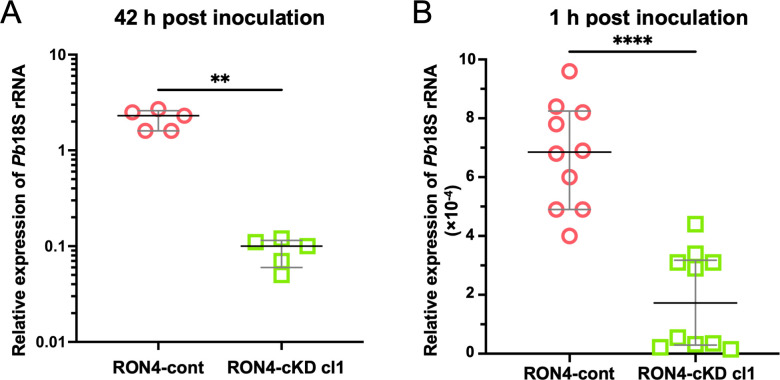
RON4 is required for sporozoite infection of the liver. Infectivity of RON4-repressed sporozoites in mice. Thirty thousand RON4-cont or RON4-cKD cl1 sporozoites were collected from salivary glands at days 20 to 23 post-feeding and were inoculated into C57BL/6 mice. Their livers were collected to measure parasite 18S rRNA levels 42 hours (**A**) or 1 hour (**B**) after inoculation. The relative parasite 18S rRNA levels were normalized to mouse *gapdh* mRNA level and are shown as dot plots. The median values of five mice (long line) are shown with the interquartile range as error bars (short bars). The statistical difference in the relative expression of *Pb*18S rRNA between RON4-cont and RON4-cKD cl1 was calculated by the Mann–Whitney *U* test (**P < 0.01, ****P < 0.0001).

At first, we clarified whether RON4 is required for sporozoite infection of the C57BL/6 mouse liver *in vivo* by intravenous injection of RON4-cont or two independent clones of RON4-cKD (cl1 and cl2) sporozoites collected from salivary glands. The relative *P. berghei* 18S ribosomal RNA (*Pb*18S rRNA) levels in the liver at 42 hours after sporozoite inoculation, when most hepatic merozoites are formed but remain inside cells, were approximately 23-fold and 14-fold lower for RON4-cKD cl1 and RON4-cKD cl2, respectively, compared to that of livers inoculated with RON4-cont sporozoites (Fig. 3A and Fig. S4A) This result showed that RON4 has crucial roles during sporozoite infection of the liver *in vivo*.

Sporozoites in the bloodstream cross the sinusoidal cell layer to infect hepatocytes, which is completed within 1 hour after inoculation (33). To determine whether RON4 is required for sporozoite crossing the sinusoidal cell layer *in vivo*, sporozoite relative amounts in the liver after perfusion at 1-hour post-inoculation of 30,000 salivary gland–derived sporozoites were compared between RON4-cont and RON4-cKD by real-time RT-PCR. The relative *Pb*18S rRNA levels of RON4-cKD cl1 and RON4-cKD cl2 in the liver were approximately 4.0-fold and 4.5-fold less, respectively, than that of RON4-cont sporozoites (Fig. 3B and Fig. S4B). This result demonstrates that RON4 knockdown decreases sporozoite accumulation in the liver parenchyma, likely due to a compromised ability to cross the sinusoidal cell layer. In addition, no parasites reappeared in the C57BL/6 mouse blood after 2,000 RON4-cKD sporozoites were intravenously inoculated, while all mice were infected by inoculation of the same amount of RON4-cont sporozoites (Fig. S5). Taken together, RON4 is important for sporozoites to exit from the bloodstream, in addition to the infection of the liver.

### RON4 is required for sporozoite cell traversal ability and hepatocyte invasion efficacy

**Fig 4:**
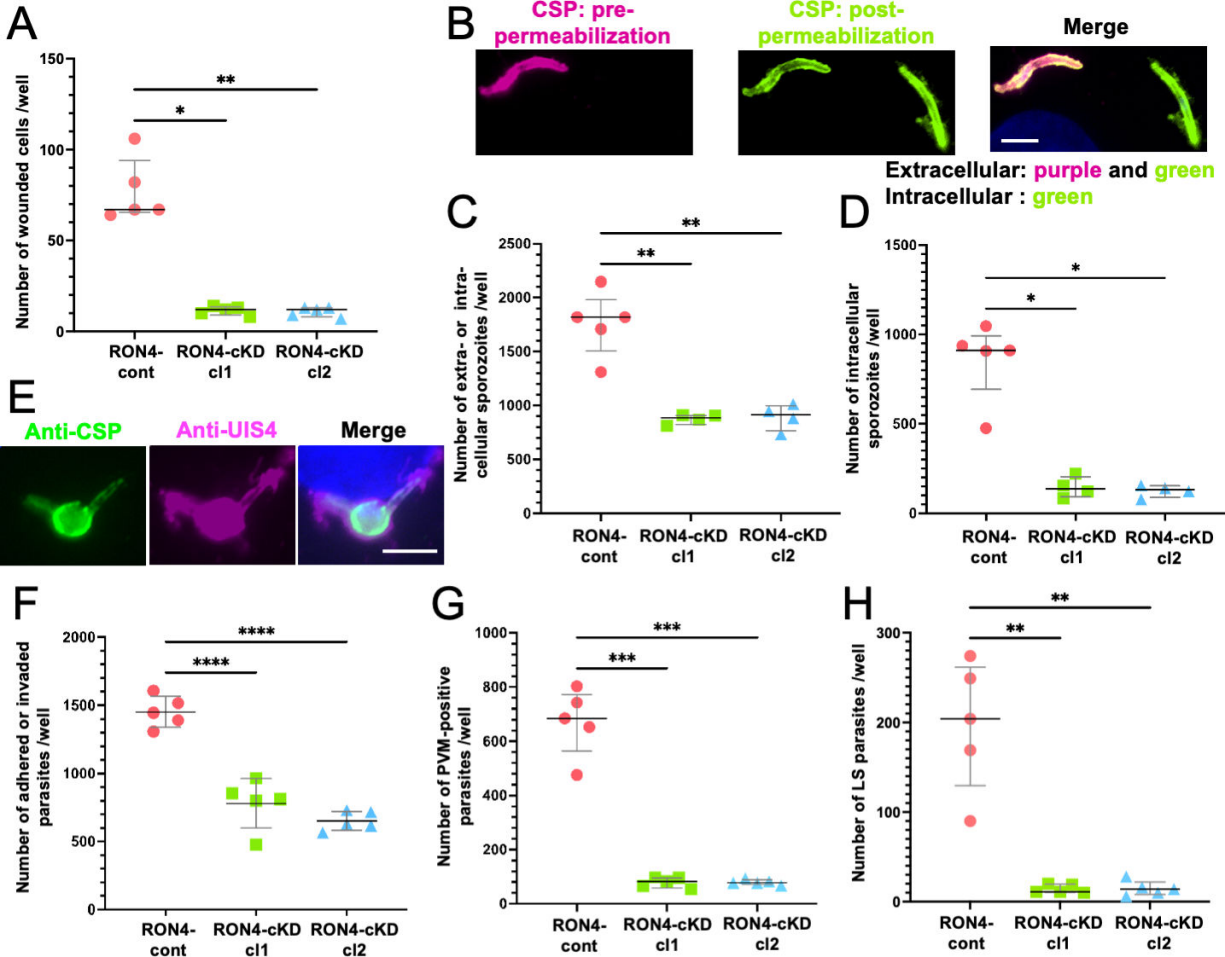
RON4 is required for sporozoite ability to traverse cells and invade hepatocytes. (**A**) The cell traversal ability of sporozoites was examined by a cell wounding assay using fluorescein-conjugated dextran. Ten thousand hemolymph sporozoites were collected from RON4-cont, RON4-cKD cl1, or RON4-cKD cl2 infected mosquitoes and inoculated onto 3T3 cells cultured confluently in eight-well chamber slides. Cells were incubated for 1 hour with 2 mg/mL of fluorescein-conjugated dextran (10,000 mol wt) in RPMI 1640 containing 10% FCS. The numbers of cells harboring dextran per well are shown as dot plots. The median values of five independent experiments (long line) are shown with the interquartile range as error bars (short bars). The statistical difference was calculated by Kruskal–Wallis followed by Dunn’s *post hoc* test (*P < 0.05, **P < 0.01). (**B–H**) The invasion ability of RON4-cKD sporozoites to HepG2 was examined *in vitro*. RON4-cont, RON4-cKD cl1, or RON4-cKD cl2 sporozoites (10,000 **[B–D**] or 5,000 **[E–H**]) were collected from salivary glands and inoculated onto HepG2 cells cultured in an eight-well chamber slide and then incubated for 1 hour (**B–D**), 6 hours (**E–G**), or 48 hours (**H**) at 37°C. The numbers of extracellular or intracellular sporozoites (**C**), intracellular sporozoites (**D**), adhered or invaded parasites (**F**), invaded parasites with PVM (**G**), and LS parasites (**H**) are shown as dot plots. The median (C, D, G, and H) or mean (**F**) values of five independent experiments (long line) are shown with the interquartile range (C, D, G, and H) or SD as error bars (**F**) (short bars). Experiments were repeated more than four times with more than three wells for each parasite line. The statistical difference was calculated by the Brown–Forsythe test followed by Dunnett’s *post hoc* test (C, G, and H), Kruskal–Wallis followed by Dunn’s *post hoc* test (**D**), or one-way ANOVA followed by Dunnett’s multiple comparison test (**F**). *P < 0.05, **P < 0.01, ***P < 0.001, ****P < 0.0001. (**B**) Representative IFA images of adhered and invaded sporozoites at 1 hour after inoculation. Sporozoites were stained by anti-CSP antibodies pre- (purple) and post- (green) permeabilization of cells. Nuclei are visualized with DAPI in merged images (blue). An extracellular sporozoite is visualized by purple and green fluorescence (merged panel, left sporozoite), while an intracellular sporozoite is visualized only by green fluorescence (merged panel, right sporozoite). Bar, 5 µm. (**E**) The parasites were detected by immunofluorescence using antibodies against UIS4 (magenta, middle panel) and CSP (green, left panel). Nuclei are visualized with DAPI in merged images (blue, right panel). Bar, 5 µm.

It was reported that to cross the sinusoidal cell layer, sporozoites need to penetrate cells by wounding the cellular membrane, called cell traversal ability (29). To investigate the cell traversal ability of salivary gland sporozoites, we performed *in vitro* cell wounding assays by detecting dextran-fluorescein isothiocyanate-containing cells due to membrane damage by sporozoite penetration. The cell traversal ability of RON4-cKD cl1 and cl2 sporozoites, indicated by the number of wounded cells, was significantly reduced to 18% of RON4-cont parasites (Fig. 4A). These results confirmed that RON4 contributes to the sporozoite cell traversal ability, which is required for crossing the sinusoidal cell layer to arrive at the liver parenchyma.

To examine whether RON4 is involved in sporozoite invasion of hepatocytes, an *in vitro* sporozoite invasion assay was performed. One hour after sporozoite inoculation onto HepG2 cells, extracellular or intracellular sporozoites were distinguished by double staining using anti-CSP mouse and rabbit antibodies (30) (Fig. 4B). For RON4-cont parasites, greater than 18% of inoculated sporozoites were detected as adhered to or invaded HepG2 cells at 1-hour post-inoculation, and approximately half of them were located intracellularly (Fig. 4C and D; Table 1). For RON4-cKD cl1 and cl2, roughly 9% of inoculated sporozoites were detected extra- or intracellularly, demonstrating that their adhesion ability decreased to approximately half of the control. In addition, only 15% and 14% of these attached RON4-cKD cl1 and cl2 sporozoites invaded HepG2 cells, respectively, indicating that RON4 repression also reduces sporozoite invasion efficiency after adhesion of cells to about 30% of the control (Fig. 4C and D; Table 1). As a result, roughly 1% of inoculated RON4-cKD sporozoites successfully invaded hepatocytes, while 9% of RON4-cont sporozoites invaded cells (Fig. 4D and Table 1). These results indicate that RON4 is involved in sporozoite adhesion to and invasion of hepatocytes.

**TABLE 1:** The number and percentage of extracellular or intracellular sporozoites 1 hour after inoculation

	Inoculated	Extra- or intracellular		Intracellular		Ratio of intracellular sporozoites/detected sporozoites (%)
		Median (Min.–Max.)^*a*^, % inoculated^*b*^	% RON4-cont	Median (Min.–Max.)^*a*^, % inoculated^*b*^	% RON4-cont	
RON4-cont	10,000	1,819 (1,308–2,147), 18.2%	100	909 (476–1,045), 9.1%	100	50
RON4-cKD cl1	10,000	887 (809–907), 8.9%	48.8	136 (82–220), 1.4%	15	15.3
RON4-cKD cl2	10,000	913 (730–1,011), 9.1%	50.2	131 (77–155), 1.3%	14.4	14.3

^*a*^ “Extra- or intracellular” and “Intracellular”: median, minimum, and maximum values in parentheses.

^*b*^ “Extra- or intracellular” and “Intracellular”: percentage of inoculated sporozoites.

To examine if the invaded sporozoites are surrounded by a PVM, sporozoite-inoculated HepG2 cells were stained with anti-UIS4 antibodies, a PVM marker, together with anti-CSP antibodies (Fig. 4E). The numbers of detected RON4-cKD sporozoites, which included both adhered and invaded sporozoites, were reduced to about 50% of that of the RON4-cont, which confirmed that RON4 repression decreased sporozoite attachment ability (Fig. 4F). Approximately 7% of RON4-cont inoculated parasites were surrounded by PVM, whereas 0.8% each of RON4-cKD cl1 and cl2 parasites were within PVM at 6 hours after sporozoite inoculation (Fig. 4G and Table 2). Taking into consideration that the ratios of RON4-cKD parasites surrounded by PVM compared to RON4-cont cl1 and cl2 were approximately 12% and 11%, which were comparable to the ratio of RON4-cKD invaded sporozoites at 1-hour post-inoculation, it indicated that the major RON4 function takes place at the early stage of hepatocyte infection, that is, adhesion to and invasion of hepatocytes (Table 1 and 2).

**TABLE 2:** The number and percentage of adhered or invaded parasites 6 hours after inoculation

	Inoculated	Adhered or invaded		Invaded (PVM positive)	
		Median (Min.–Max.)^***a***^, % inoculated^***b***^	% RON4-cont	Median (Min.–Max.)^***a***^, % inoculated^***b***^	% RON4-cont
RON4-cont	10,000	1,445 (1,308–1,516), 14.5%	100	685 (476–802), 6.9%	100
RON4-cKD cl1	10,000	809 (477–966), 8.1%	56	82 (53–96), 0.8%	12
RON4-cKD cl2	10,000	629 (565–730), 6.3%	43.5	77 (66–94), 0.8%	11.2

^*a*^ “Adhered or invaded” and “Invaded”: median, minimum and maximum values in parentheses.

^*b*^ “Adhered or invaded” and “Invaded”: percentage of inoculated sporozoites.

**TABLE 3:** The proportion of sporozoites showing each attachment and motility pattern (%)

Sporozoite attachment and motility pattern (%)	Full-length-attached and motility (+) ^*a*^	Full-length-attached and motility (−) ^*a*^	Partial-attached ^*a*^	Non-attached ^*a*^
RON4-cont	61.5 (50.8-80.3)	0.9 (0.0-2.3)	8.2 (0.3-22.9)	25.7 (11.6-35.0)
RON4-cKD cl1	9.2 (0-13.7)	14.9 (4.4-18.6)	22.7 (12.0-25.7)	55.2 (45.7-83.5)
RON4-cKD cl2	10.0 (1.7-13.4)	15.4 (8.7-23.5)	20.9 (14.8-35.0)	50.0 (43.6-72.6)

^*a*^ Median, minimum, and maximum values in parentheses.

The importance of RON4 during sporozoite infection of hepatocytes was further confirmed by continuous incubation until 48-hour post-sporozoite inoculation, a time at which LS parasites grow sufficiently to be easily detected by IFA using antibodies against CSP and UIS4 (34). The LS parasite numbers were decreased in RON4-cKD cl1 and cl2 parasites to approximately 19-fold and 15-fold less, respectively, compared to RON4-cont parasites (Fig. 4H). This result was consistent with a study using the Cre/LoxP-dependent gene excision conditional knockout system (24). The diameters of RON4-cKD LS parasites were comparable to those of RON4-cont (Fig. S6), indicating that RON4 is dispensable for LS development after sporozoites invade hepatocytes. Taken together, *in vitro* assays demonstrated that RON4 is crucial for both cell invasion pathways, specifically, traversing cells by membrane wounding and invading hepatocytes with PVM formation.

### RON4 has important roles in sporozoite attachment and motility

**Fig 5:**
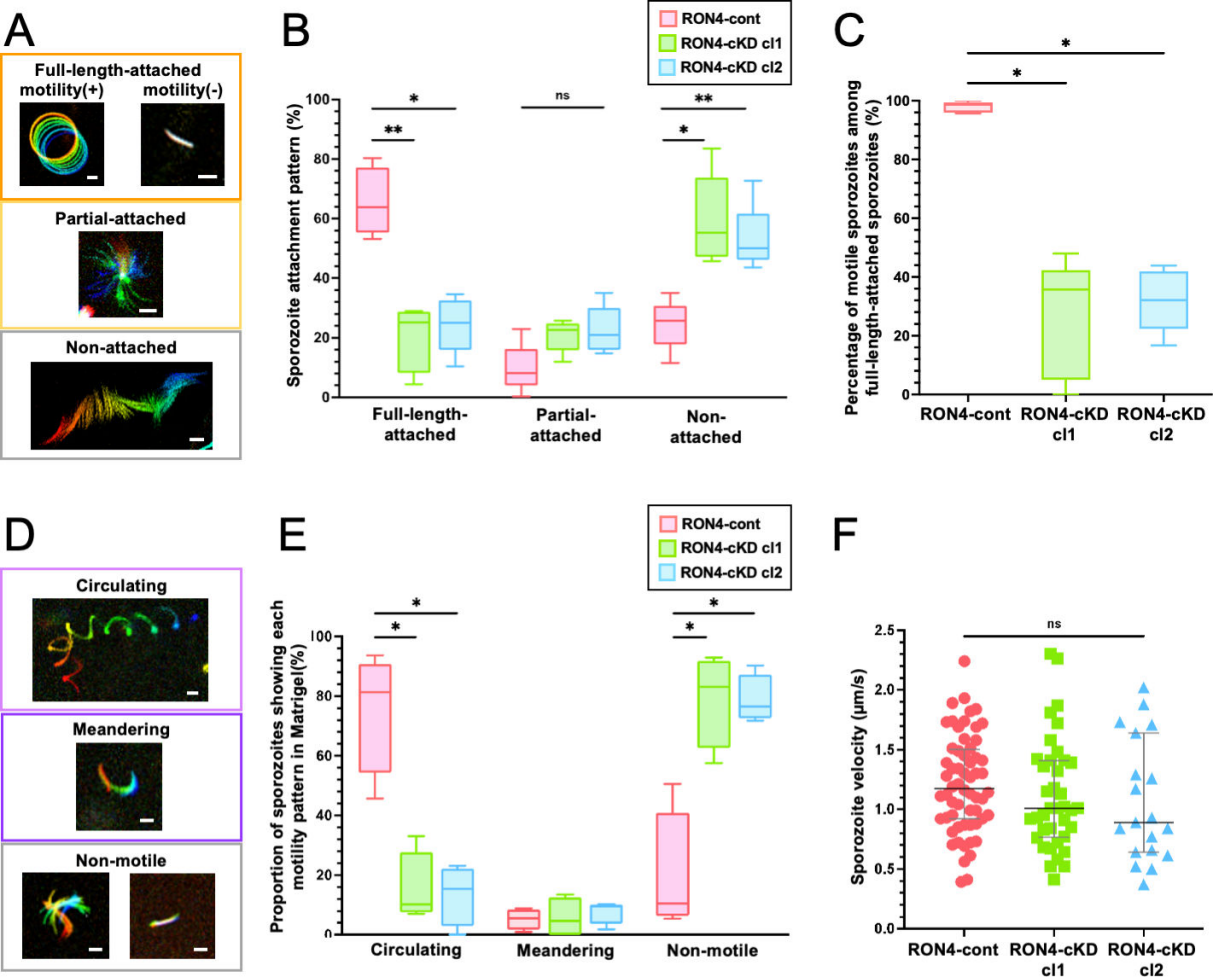
Comparison of sporozoite attachment and motility among transgenic parasite lines. The attachment and motility ability of sporozoites collected from salivary glands at days 19 to 26 post-feeding were examined *in vitro*. RON4-cont, RON4-cKD cl1 or cl2 sporozoites were inoculated onto a glass bottom dish (**A–C**) or embedded in Matrigel (**D–F**), then their movements were recorded every 2 seconds for about 5 minutes (up to 150 frames). (**A**) Time-lapse micrographs of each attachment pattern. Sporozoites were categorized as full-length-attached (upper panel), partial-attached (middle panel), and non-attached (lower panel). Full-length-attached sporozoites were further categorized depending on their motility. Bars, 5 µm. (**B**) Sporozoite attachment patterns were compared between RON4-cont and RON4-cKD sporozoites. Box plots demonstrate the percentage of sporozoites showing each attachment pattern with error bars indicating the minimum value and maximum value from five independent experiments containing at least 80 sporozoites per parasite line (red: RON4-cont, green: RON4-cKD cl1, blue: RON4-cKD cl2). The statistical difference in each attachment pattern was calculated by Kruskal–Wallis followed by Dunn’s *post hoc* test (*P < 0.05, **P < 0.01, ns: no statistical difference). (**C**) The percentage of motile sporozoites in full-length-attached sporozoites. Box plots demonstrate the percentage of gliding sporozoites with error bars indicating minimum value and maximum value. Experiments were repeated five times with at least 110 (RON4-cont) or 25 (RON4-cKD cl1 and cl2) sporozoites per parasite line. The statistical difference was calculated by Kruskal–Wallis followed by Dunn’s *post hoc* test (*P < 0.05). (**D**) Time-lapse micrographs of sporozoite motility patterns in Matrigel. Sporozoites were categorized as circulating (upper panel), meandering (middle panel), and non-motile (lower panel). Bars, 5 µm. (**E**) The box plots show the percentages of sporozoites showing each motility pattern with minimum and maximum values as error bars from four independent experiments with at least 40 sporozoites per parasite line (red: RON4-cont, green: RON4-cKD cl1, blue: RON4-cKD cl2). The statistical difference in each attachment pattern was calculated by Kruskal–Wallis followed by Dunn’s *post hoc* test (*P < 0.05). (**F**) The velocity of each circulating sporozoite (*n* > 19) was plotted. Sporozoite movement was traced using the MTrack2 plugin in Fiji software. No significant difference in the velocity was detected among RON4-cont, RON4-cKD cl1, and RON4-cKD cl2 examined by the Kruskal–Wallis test (*P* = 0.18).

The attachment ability and motility of sporozoites have been shown to be essential for the infection of the liver (20, 35 - 37). We have reported that RON4-cKD sporozoites collected from mosquito hemolymph show less attachment ability and motility than RON4-cont sporozoites, which results in less invasion efficiency to salivary glands (21). Here, we assessed the *in vitro* gliding ability of RON4-cKD sporozoites that had successfully invaded salivary glands. Sporozoites were incubated with FCS containing medium on glass slides and categorized depending on their attachment patterns as follows: full-length-attached, partial-attached, and non-attached (Fig. 5A). The population of full-length-attached sporozoites decreased in RON4-cKD cl1 and cl2 approximately three-fold compared with the RON4-cont (Fig. 5B; the median ratio was 64% in RON4-cont and 25% in RON4-cKD). Accordingly, the population of non-attached sporozoites increased in RON4-cKD cl1 and cl2 approximately two-fold compared with the RON4-cont (Fig. 5B; the median ratio was 26% in RON4-cont, 55% in RON4-cKD cl1, and 50% in RON4-cKD cl2). Taken together, RON4 is involved in the attachment ability of sporozoites residing in salivary glands, similar to sporozoites collected from the hemolymph.

Next, RON4 contributions to sporozoite motility were analyzed. Among full-length-attached sporozoites, over 95% of sporozoites in RON4-cont showed gliding motility versus 36% and 32% in RON4-cKD cl1 and cl2, respectively (Fig. 5C). As a result, the proportions of gliding sporozoites in inoculated sporozoites were 62% in RON4-cont, 9% in RON4-cKD cl1, and 10% in RON4-cKD cl2 (Table 3), indicating that RON4 is important for both attachments to the glass slide and motility.

To confirm whether RON4 is required for sporozoite motility as well as attachment ability, sporozoites were embedded in Matrigel, a matrix composed of laminin and type IV collagen, thus bypassing the adhesion step to the substrate. Sporozoite-moving patterns were analyzed by the categories described by Nozaki et al. (21), as follows: circulating, continuous migration through the Matrigel in a circular pattern; meandering, incompletely proceeding; and non-motile, not moving forward or staying immotile (Fig. 5D). When embedded in Matrigel, 81% of RON4-cont sporozoites circulated, whereas only 10% and 16% of RON4-cKD cl1 and cl2 sporozoites circulated, respectively (Fig. 5E). Accordingly, 74% and 66% of RON4-cKD cl1 and cl2 sporozoites did not move forward in the Matrigel, whereas 10% of RON4-cont sporozoites remained non-motile (Fig. 5E). On the other hand, the velocity of continuously moving sporozoites showed no significant difference between RON4-cont and RON4-cKD sporozoites (Fig. 5F). These results demonstrate that RON4 in salivary gland sporozoites has important roles both in attachment to substrates and in the initiation of movement; however, it is not essential for moving speed, which is mediated by an actomyosin system called the glideosome (38). Attachment is required during gliding, and thus the duration of attachment is also reduced by RON4 repression (Fig. S7).

### Anti-RON4 antibody suppressed sporozoite invasion of hepatocytes

**Fig 6:**
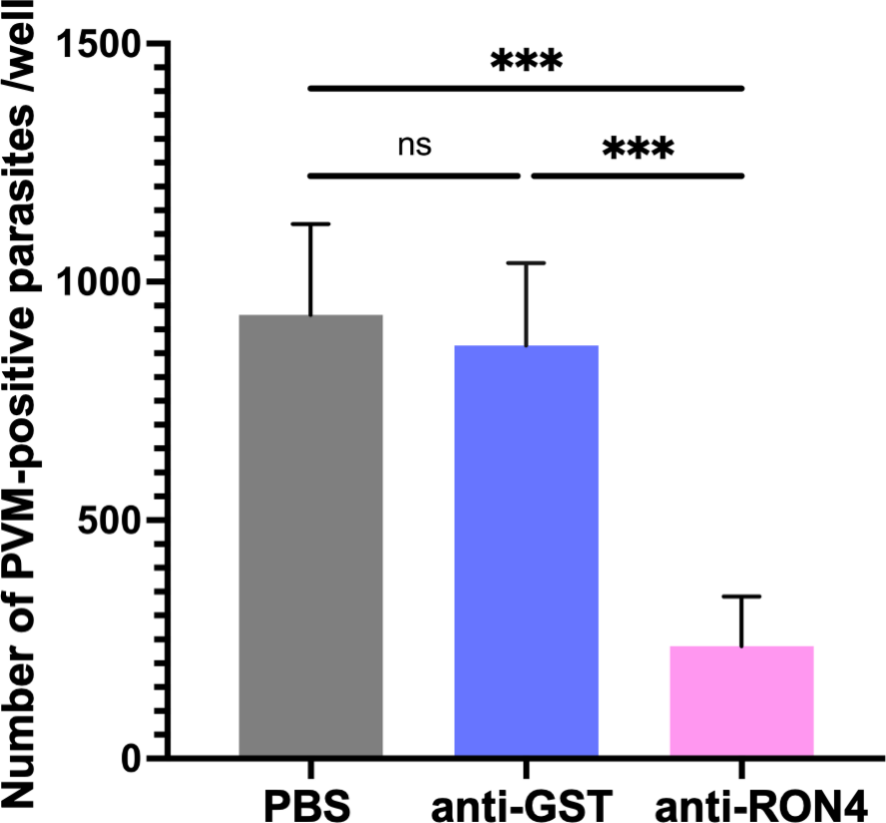
Antibodies against RON4-suppressed sporozoite infection of hepatocytes. Twenty thousand *Pb*ANKA-GFP sporozoites were collected from salivary glands and were inoculated onto HepG2 cells cultured in an eight-well chamber slide. The cells were incubated for 1.5 hours with 1 mg/mL of anti-RON4 antibody, anti-GST antibody, or PBS included in the culture medium. After 6 hours of incubation, the cells were washed with PBS, fixed with 10% formalin, and permeabilized by 0.1% Triton X-100. The parasites were detected by immunofluorescence using antibodies against UIS4 and CSP. The mean values of PVM-positive parasites are shown as bars with SD as error bars, from four experiments with three wells for each antibody treatment. The statistical difference was calculated by one-way ANOVA followed by Dunnett’s multiple comparison test (***P < 0.001, ns: no statistical difference).

Next, we investigated whether RON4 is secreted during migration and invasion of cells, as reported for *Plasmodium yoelii* sporozoites (23). RON4 could be detected at the tips of sporozoites without permeabilization by incubation in FCS-containing medium to activate gliding (Fig. S8). To confirm RON4 secretion, we examined whether anti-RON4 antibodies inhibit sporozoite infection of hepatocytes. Sporozoites were isolated from salivary glands, and prior to inoculation HepG2 cells were incubated with antibodies against RON4 or GST as a negative control. The invaded parasites surrounded by PVM were reduced to approximately 30% of control by anti-RON4 antibody treatment (Fig. 6A), and inhibition of sporozoite invasion was in an antibody dose-dependent manner (Fig. S9). These results indicate that secreted RON4, which could be targeted by antibodies, functions during sporozoite infection of hepatocytes.

## DISCUSSION

In this study, we have demonstrated *in vivo* that RON4 is important for *Plasmodium* sporozoite transmission from the mosquito to the mammalian liver, in addition to sporozoite invasion of mosquito salivary glands (21). Using RON4-cKD sporozoites generated by the promoter substitution method, we revealed that RON4 has important roles in cell traversal, which is required for migration through the sinusoidal cell layer to reach hepatocytes, and in hepatocyte invasion with PVM formation. After hepatocyte invasion, RON4 ceases expression and does not appear to be involved in LS parasite development or differentiation into liver merozoites.

These results confirmed studies that used conditional RON4-knockout sporozoites by inducible FLP-FRT recombination and DiCre excision systems, demonstrating that RON4 is required for hepatocyte infection *in vitro* (22, 24). Unlike these gene excision systems, in which a small portion of RON4 expressing sporozoites remained, our RON4-cKD sporozoites by promotor-swapping repressed RON4 uniformly (21). Therefore, in this study, we could use an *in vivo* infection model to address in detail further questions about the roles of RON4 during sporozoite infection of the liver.

Previously, we revealed that the RON complex components (RON2, RON4, and RON5) are involved in hemolymph sporozoite motility, which is required for sporozoite invasion of salivary glands (20, 21). Since motility is also known to be crucial for sporozoite transmission from mosquitoes to mammalian livers, we investigated the contribution of RON4 to these processes. This study revealed that RON4 in salivary gland sporozoites is important for attachment ability, which is crucial for multiple steps such as the initiation and continuation of sporozoite migration (Fig. 5B and C; Fig. S7). In addition, motility did not recover even after RON4-cKD sporozoites were embedded in Matrigel (Fig. 5E), revealing that RON4 is not only important for sporozoite attachment prior to gliding but also for the initiation of gliding. Considering a previous report demonstrating that the turnover of discrete adhesion sites is crucial for the continuous movement of sporozoites (39), RON4 is required for sporozoite migration via mediating adhesion ability.

Motility is essential for sporozoites deposited by the mosquito into the skin to migrate and infect hepatocytes *in vivo* (40) and is dependent on the secretion of microneme proteins, such as TRAP (41, 42). Studies on micronemal proteins demonstrated that mutant sporozoites with less attachment/gliding ability show decreased cell traversal ability (43 - 46), which is required for crossing the sinusoidal cell layer (29). Such a phenotype was observed for the TRAP-like protein (TLP) knockout, *Plasmodium* protein *O*-fucosyltransferase (POFUT2) knockout, and the Cell Traversal for Ookinetes and Sporozoites (S4/Celtos) knockdown. RON4-cKD sporozoites also showed phenotypes of both a decreased ability in cell traversal *in vitro* and in crossing the sinusoidal cell layer *in vivo* (Fig. 3B, Fig. 4A, and Fig. S4B), possibly due to reduced attachment and initiation of gliding. As discussed above, it seems reasonable that the traversal ability of sporozoites depends on their attachment ability, because continuous migration requires the turnover of adhesive sites. However, further confirmation is necessary; for example, using different cultured cells and detection methods could fill the gap in the results of the cell traversal assays between Fig. 4A and Fernandes et al. (22), indicating that there was no statistical difference in cell wounding ability as detected by flow cytometry. RON2-cKD sporozoites also demonstrated reduced cell traversal ability, supporting that RON2 and RON4 function cooperatively through their interaction during migration and invasion. This is the first report to demonstrate the involvement of rhoptry proteins in attaching to and/or crossing the sinusoidal cell layer *in vivo*.

Microneme proteins are secreted from sporozoites during gliding and have been suggested to have a function in motility, such as TRAP, CSP, S4/Celtos, TRAP-related protein (S6/TREP/UOS3) (47 - 49), and LIMP (35). In contrast, it remained unclear how rhoptry proteins are involved in sporozoite attachment and motility. It was reported that RON4 is secreted during sporozoite infection of hepatocytes (23, 50). Furthermore, in *Toxoplasma* sporozoites, RON4 was detected at the moving junction during the invasion of cells (51). In this study, RON4 secretion was also detected during gliding (Fig. S8), suggesting that secreted RON4 mediates sporozoite attachment directly. In addition, we have demonstrated that anti-RON4 antibodies suppressed sporozoite infection of hepatocytes (Fig. 6 and Fig. S9). Taken together, our study indicated that RON4 secretes and functions during gliding and infection of hepatocytes. Further investigations might address if RON4 in sporozoites functions as a complex with AMA1, a known partner protein of the RON complex in merozoites.

In consideration of RON4 expression and its crucial roles both in *Plasmodium* sporozoites and merozoites, RON4 and other secreted rhoptry proteins might be new targets for the development of vaccines that could recognize both infectious stages in the *Plasmodium* life cycle. This possibility is supported by field-based reports suggesting that an anti-RON4 antibody titer is positively linked to malarial protection (52, 53).

It has been revealed that the rhoptry proteins RON4, RON2, RON5, and RON11 are crucial for sporozoite invasion via mediating attachment ability and motility (20, 21, 30). Further analyses on the secretion mechanism and interaction of these proteins, plus integration with the knowledge of micronemal proteins related to sporozoite gliding, will contribute to elucidate the comprehensive mechanisms of sporozoite infection.

## SUPPLEMENTAL MATERIAL

Figure S1 msphere.00587-22-s0001.tiff Generation of mCherry-tagged RON4 expressing transgenic parasites.

Figure S2 msphere.00587-22-s0002.tiff RON4 is not expressed during parasite development in hepatocytes.

Figure S3 msphere.00587-22-s0003.tif Western blot analysis of RON4 in salivary gland sporozoites.

Figure S4 msphere.00587-22-s0004.tiff RON4 is required for sporozoite infection of the liver.

Figure S5 msphere.00587-22-s0005.tiff Blood stage parasitemia after sporozoite injection.

Figure S6 msphere.00587-22-s0006.tiff RON4-cKD parasites develop normally after invasion of hepatocytes.

Figure S7 msphere.00587-22-s0007.tiff Number of gliding cycles.

Figure S8 msphere.00587-22-s0008.tif RON4 were detected at the tip of sporozoites without permeabilization.

Figure S9 msphere.00587-22-s0009.tiff Antibodies against RON4 inhibited sporozoite infection of hepatocytes in a dose-dependent manner.

TABLE S1 msphere.00587-22-s0010.pdf Table S1 and supplemental figure legends.

ASM does not own the copyrights to Supplemental Material that may be linked to, or accessed through, an article. The authors have granted ASM a non-exclusive, world-wide license to publish the Supplemental Material files. Please contact the corresponding author directly for reuse.
