# Supplementary figures and images for: Rhoptry neck protein 4 plays important roles during Plasmodium sporozoite infection of the mammalian liver
Source: mSphere. 2023 Jun 5;8(4):e00587-22. doi: 10.1128/msphere.00587-22 (PMC10449513; doi:10.1128/msphere.00587-22)

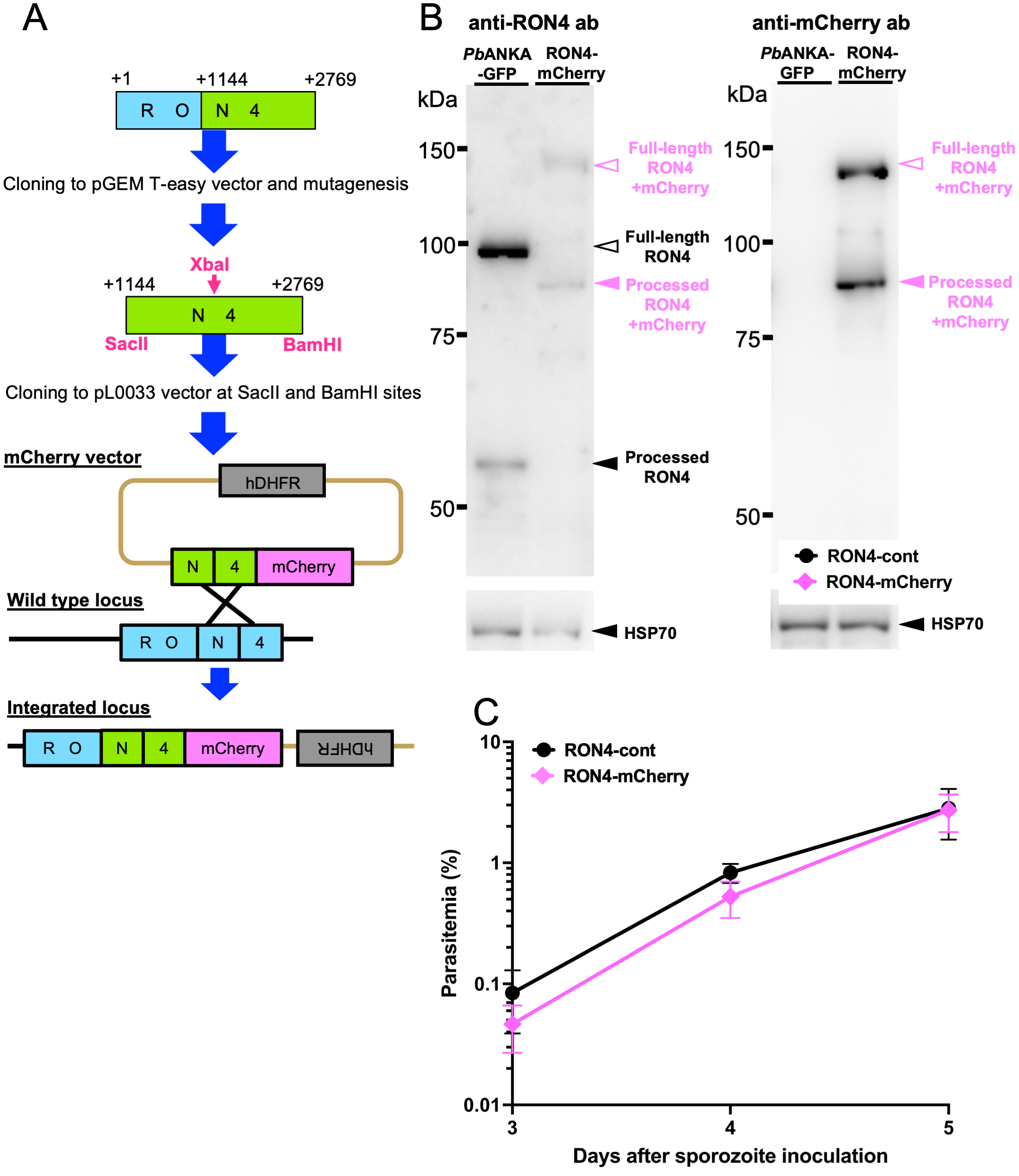

Supplement: Figure S1 — Generation of mCherry-tagged RON4 expressing transgenic parasites. [file msphere.00587-22-s0001.tiff]

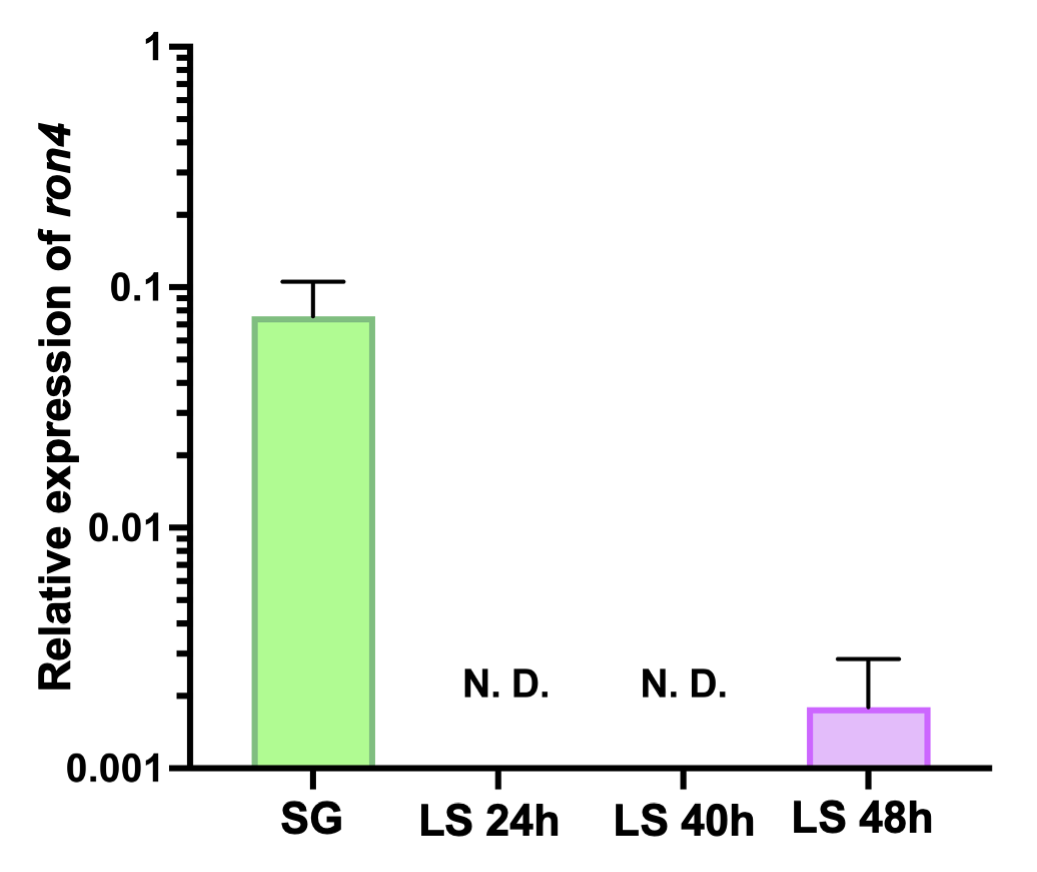

Supplement: Figure S2 — RON4 is not expressed during parasite development in hepatocytes. [file msphere.00587-22-s0002.tiff]

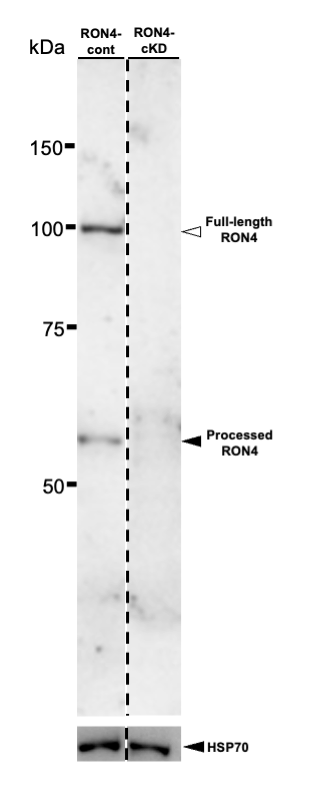

Supplement: Figure S3 — Western blot analysis of RON4 in salivary gland sporozoites. [file msphere.00587-22-s0003.tif]

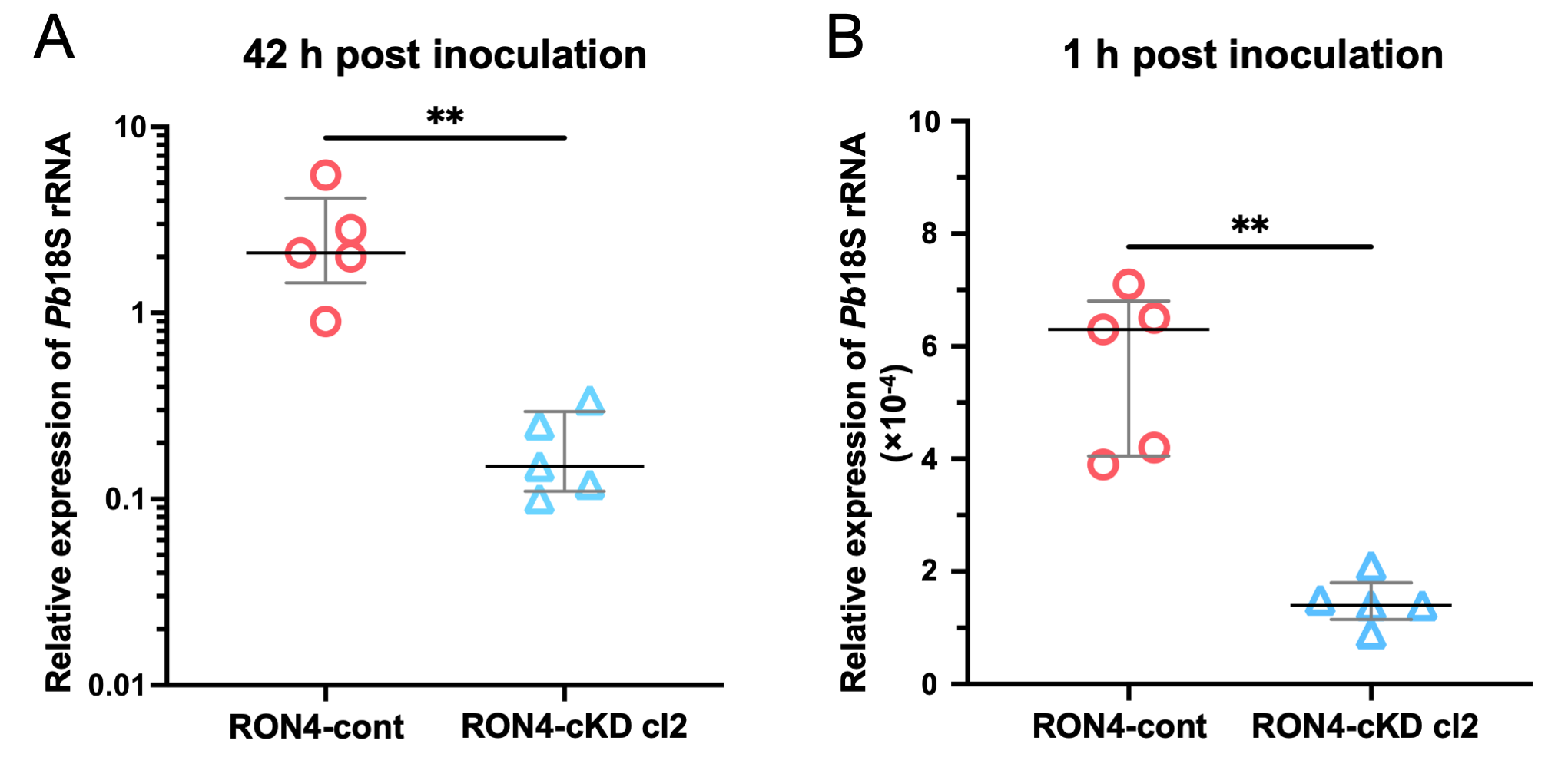

Supplement: Figure S4 — RON4 is required for sporozoite infection of the liver. [file msphere.00587-22-s0004.tiff]

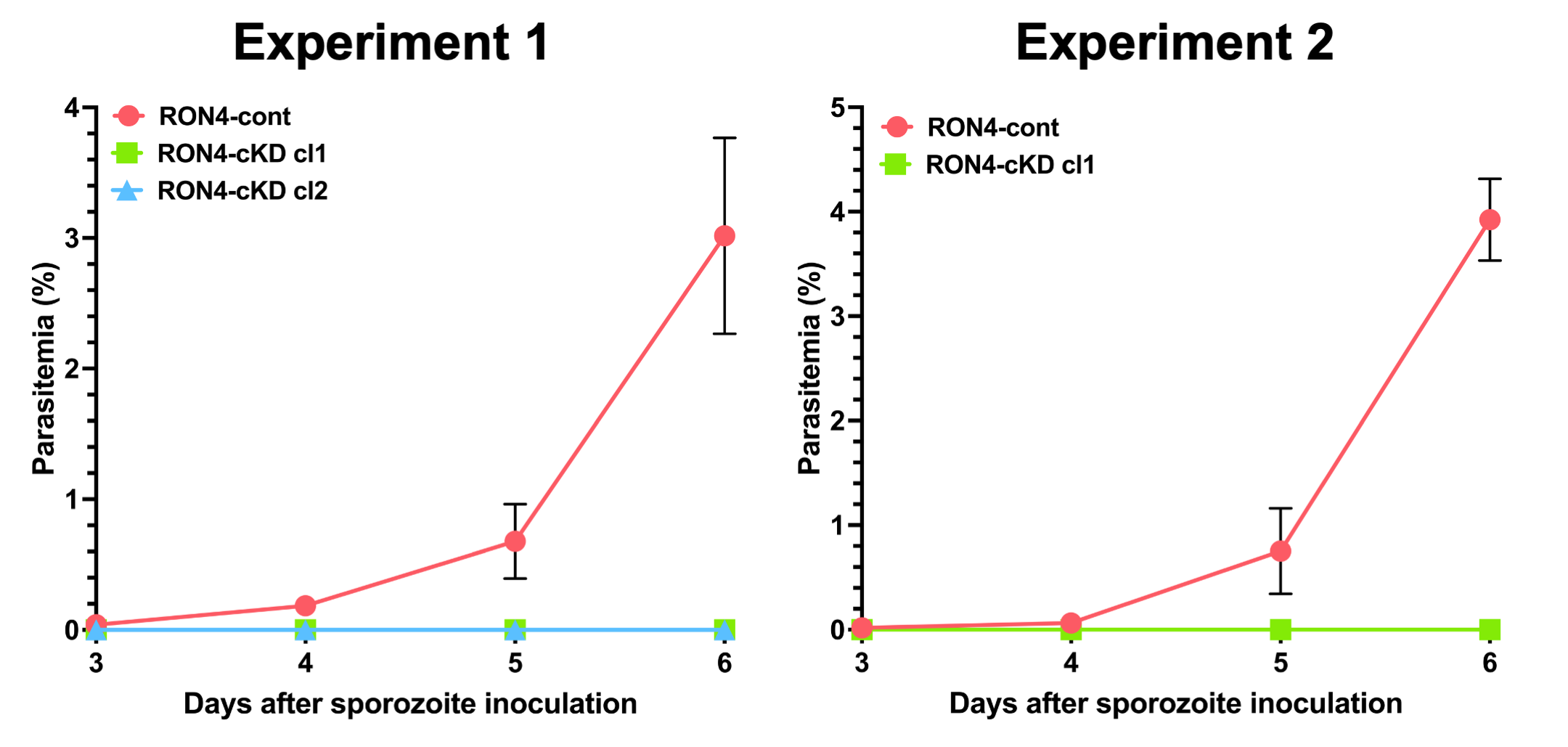

Supplement: Figure S5 — Blood stage parasitemia after sporozoite injection. [file msphere.00587-22-s0005.tiff]

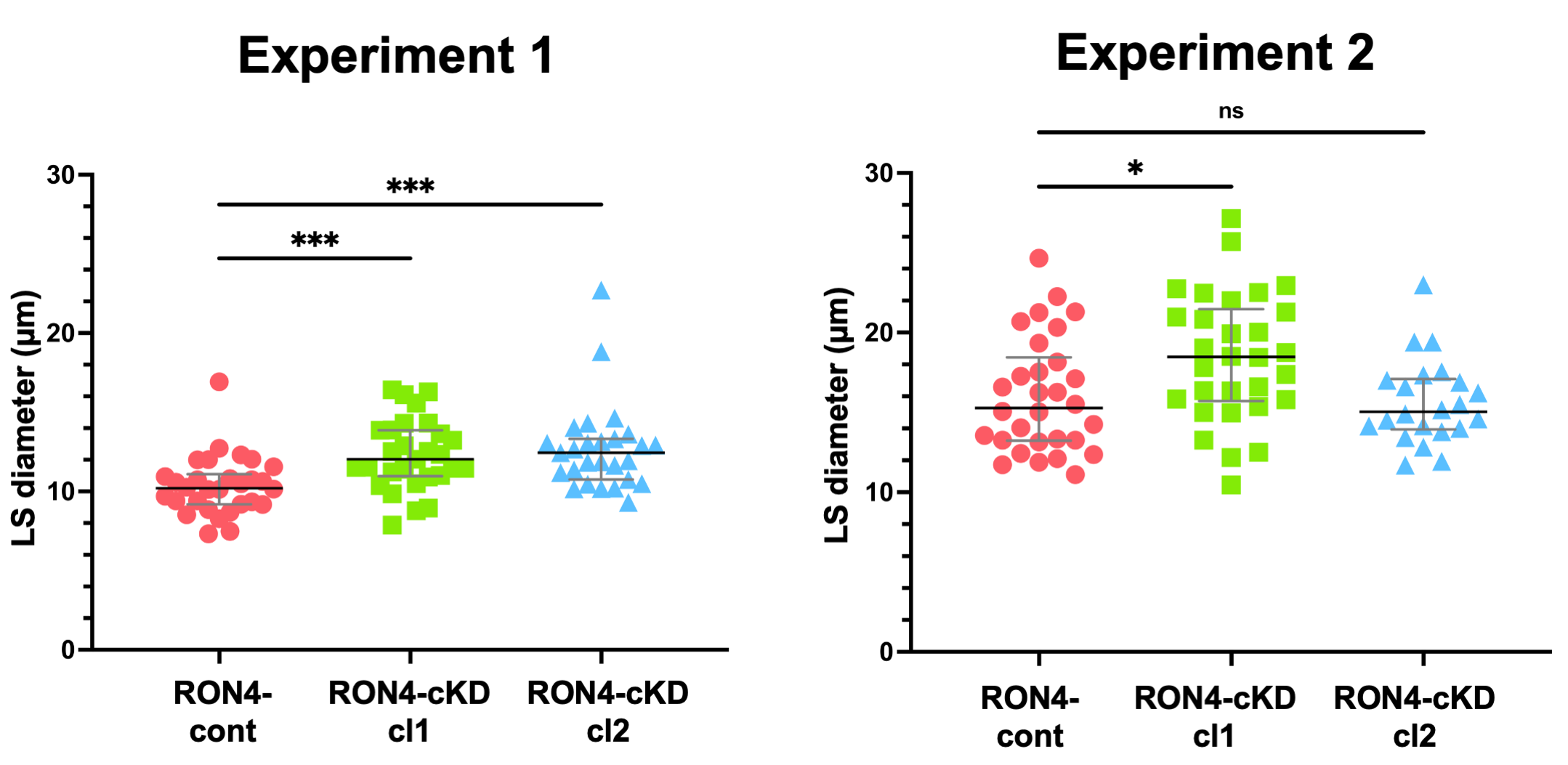

Supplement: Figure S6 — RON4-cKD parasites develop normally after invasion of hepatocytes. [file msphere.00587-22-s0006.tiff]

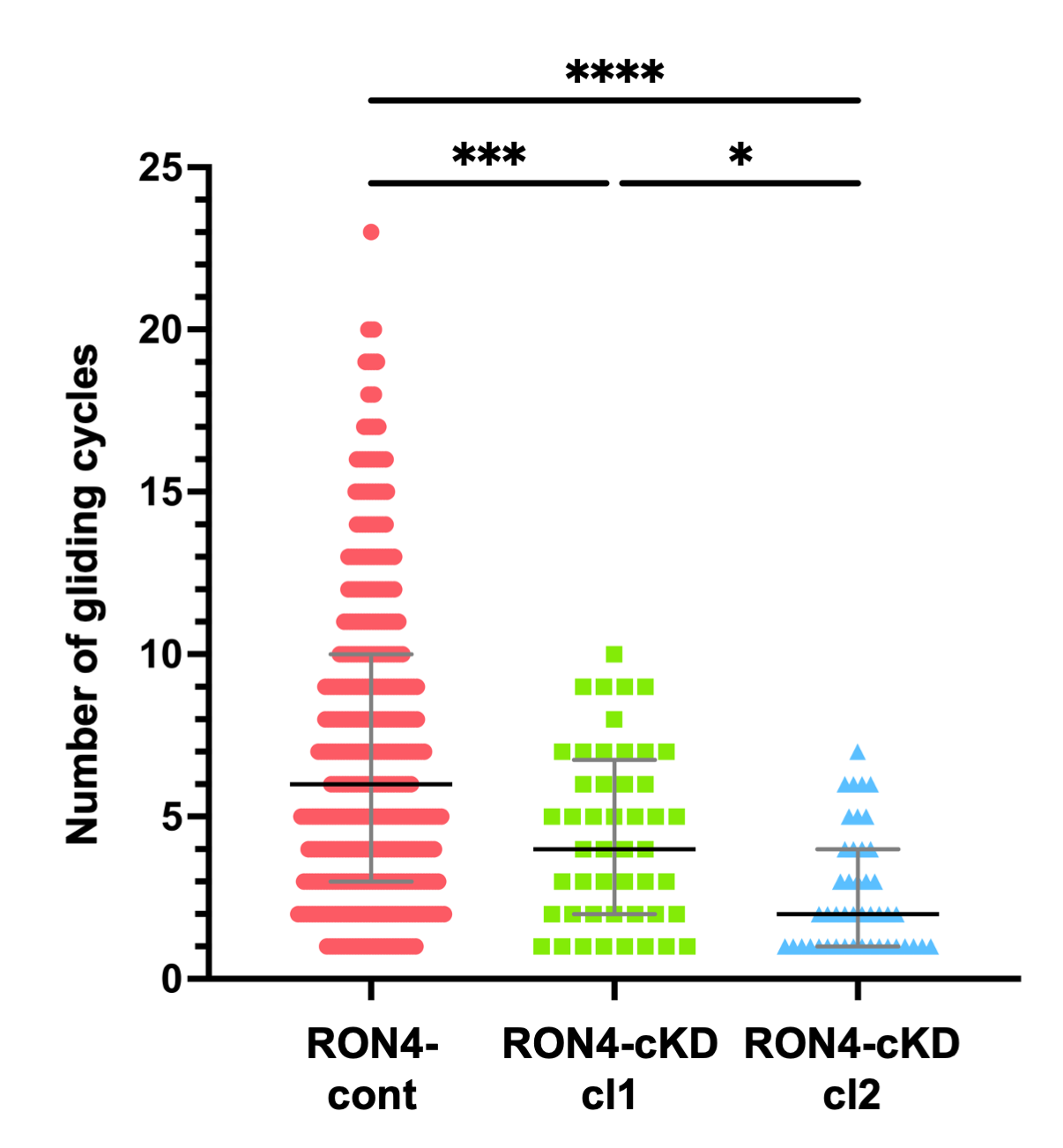

Supplement: Figure S7 — Number of gliding cycles. [file msphere.00587-22-s0007.tiff]

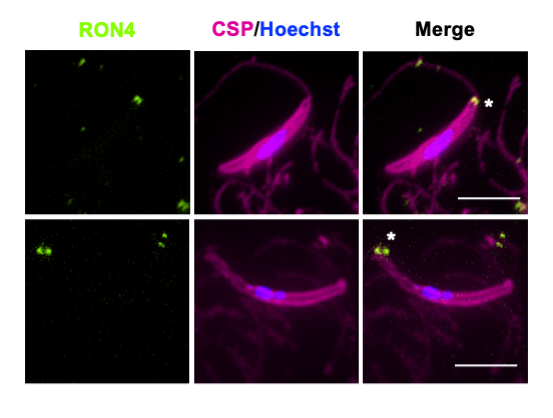

Supplement: Figure S8 — RON4 were detected at the tip of sporozoites without permeabilization. [file msphere.00587-22-s0008.tif]

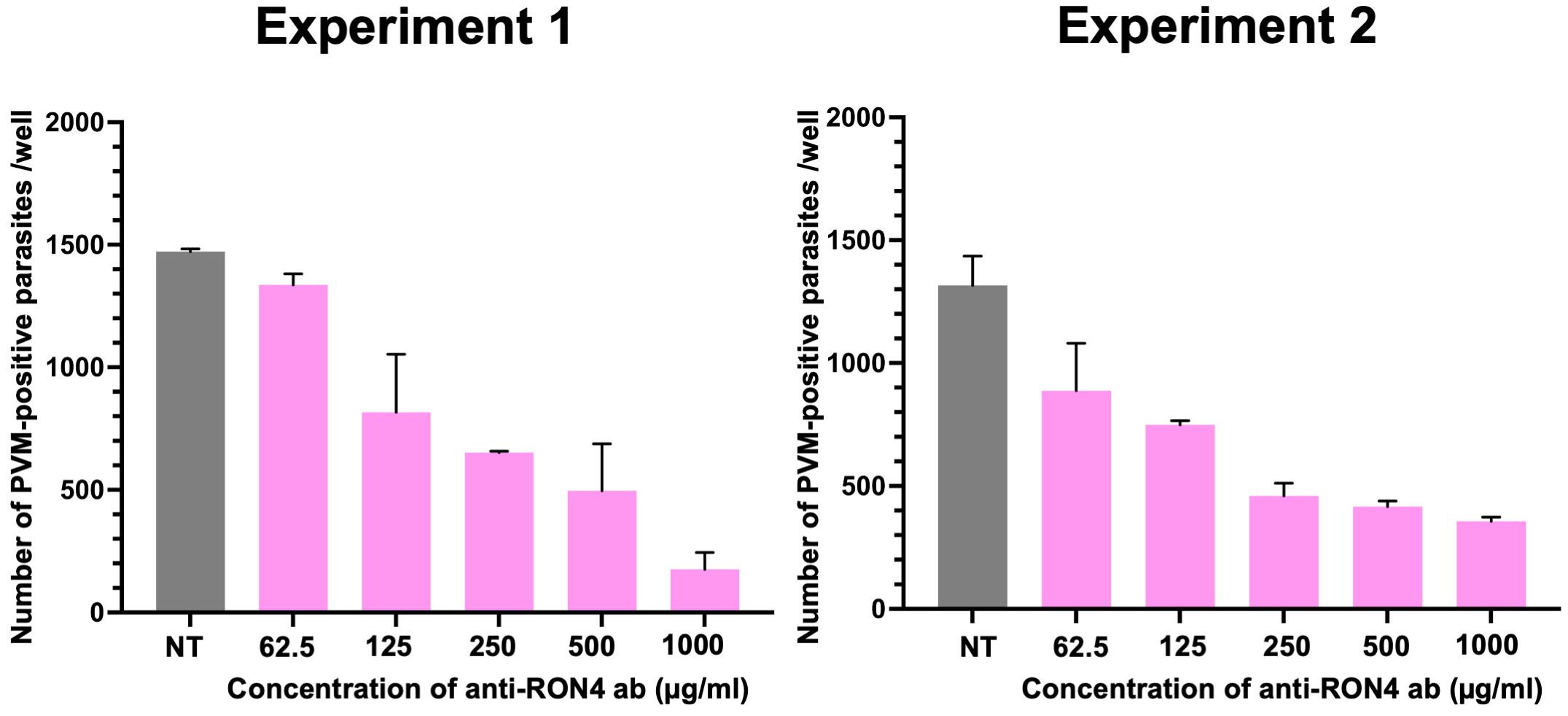

Supplement: Figure S9 — Antibodies against RON4 inhibited sporozoite infection of hepatocytes in a dose-dependent manner. [file msphere.00587-22-s0009.tiff]
