# Supplementary material for: Rhoptry neck protein 4 plays important roles during Plasmodium sporozoite infection of the mammalian liver
Source: mSphere. 2023 Jun 5;8(4):e00587-22. doi: 10.1128/msphere.00587-22 (PMC10449513; doi:10.1128/msphere.00587-22)
Supplement: TABLE S1 — and supplemental figure legends. [file msphere.00587-22-s0010.pdf]

Table S1. Lists of primers used in this study.

Primer	Sequence (5‘--->3’)
**Transgenic parasite construction**	
*Pb*RON4::mCherry-F SacII	TCCccgcggGATAAAGGAATGAATAGCATTACCG
*Pb*RON4::mCherry-R BamHI	CGggatccTAAATCATCAAAAATGGCTTTCTCAAC
**Real time PCR**	
*Pb*RON4 RT-F	GCTACTTATTAGAGAGCGAAAAC
*Pb*RON4 RT-R	CATGTGCTAATACGTTGTGTG
*Pb*EF1a RT-F	TGGAACCACCCAAAAGACCA
*Pb*EF1a RT-R	ACAACAGCAGATGGAGCGAA
*Pb*18S rRNA RT-F	GGAGATTGGTTTTGACGTTTATGTG
*Pb*18S rRNA RT-R	AAGCATTAAATAAAGCGAATACATCCTTAC
*Mm*GAPDH RT-F	AGGTCGGTGTGAACGGATTTG
*Mm*GAPDH RT-R	TGTAGACCATGTAGTTGAGGTCA

Supplemental Figure legends

Fig. S1 Generation of mCherry-tagged RON4 expressing transgenic parasites.

(A) Schematic representation of single crossover homologous recombination to integrate an expression cassette of RON4 fused with an mCherry tag at the C-terminus into the native *ron4* locus. The number of nucleotides from the first methionine residue are indicated above. Plasmid vector was linearized by XbaI, which was introduced by a point mutation without amino acid substitution, and then transfected into *Plasmodium berghei* ANKA to obtain RON4-mCherry expressing transgenic parasites. (B) Western blot analysis of RON4 and RON4-mCherry in oocyst-derived sporozoites. Protein homogenates of 20,000 or 50,000 sporozoites collected from mosquito midguts at days 18 to 21 after infection with *Pb*ANKA-GFP and RON4-mCherry parasites, indicated above the membranes, were separated by SDS-PAGE. (Left) RON4 was detected as full-length (open arrowheads) and processed form (closed arrowheads) by anti-RON4 antibodies at predicted size in *Pb*ANKA-GFP sporozoites. In the RON4-mCherry lysate, both signals were shifted higher than those of the original RON4 due to adding the mCherry tag. (Right) These bands were also detected by anti-mCherry antibodies, confirming that RON4 was tagged by mCherry.

The molecular weight position of RON4-mCherry was shifted higher than that of original RON4 by adding the mCherry tag. (Lower panels) Protein loading was confirmed by detecting HSP70, indicated by black closed arrowheads. (C) Blood stage parasitemia after RON4-mCherry and RON4-cont sporozoite inoculation. Ten thousand salivary gland sporozoites of RON4-cont (black line) or RON4-mCherry (pink line) lines were inoculated intravenously to four female C57BL/6 mice. The graph shows from days 3 to 5 post-inoculation. The dot plots show the mean of 4 mice with standard deviation as error bars.

Fig. S2 RON4 is not expressed during parasite development in hepatocytes.

RON4 mRNA expression levels in sporozoite and liver stage parasites. Total RNA was extracted from parasite-infected mosquito salivary glands at day 17 post-feeding (SG). The mean values of relative mRNA amounts of *ron4*, normalized by *ef1α* mRNA expression, are plotted as bar graphs with standard deviations from three independent experiments. Total RNA was also extracted from sporozoite inoculated C57BL/6 mouse livers at 24, 40 and 48 hours after inoculation (LS 24 h, LS 40 h and LS 48 h). The mean values of relative

mRNA amounts of *ron4*, normalized by *ef1α* mRNA expression, are shown as bar graphs with standard deviations from 3 mice in each time point. N. D.: not detected.

Fig. S3 Western blot analysis of RON4 in salivary gland sporozoites.

RON4-cont or RON4-cKD sporozoites were collected from mosquito salivary glands and homogenized in SDS-buffer. Five thousand sporozoites were loaded, and RON4 and HSP70 were detected by anti-RON4 (upper panel) or anti-HSP70 (lower) antibodies. Full length (indicated by an open arrowhead) and processed forms (closed arrowhead) were detected in RON4-cont sporozoites, while these signals were under the detection level in RON4-cKD sporozoites.

Fig. S4 RON4 is required for sporozoite infection of the liver.

Infectivity of RON4 repressed sporozoites in mice. Reproducibility of Fig. 3 was confirmed using RON4-cKD cl2 sporozoites. The relative parasite 18S rRNA levels, normalized to the mouse *gapdh* mRNA level are shown as dot plots. The median values of five mice (long line) are shown with interquartile range as error bars (short bars). The statistical difference

in the relative expression of *Pb*18S rRNA between RON4-cont and RON4-cKD cl2 were calculated by the Mann–Whitney *U* test (**P < 0.01).

Fig. S5 Blood stage parasitemia after sporozoite injection.

Two thousand salivary gland sporozoites of RON4-cont, RON4-cKD cl1, or cl2 were inoculated intravenously to C57BL/6 mice. The graph shows from days 3 to 6 post-inoculation. The dot plots show the mean parasitemia from 3 mice with standard deviation. The experiment was performed twice.

Fig. S6: RON4-cKD parasites develop normally after invasion of hepatocytes.

The diameters of liver stage parasites were compared between RON4-cont, RON4-cKD cl1 and RON4-cKD cl2, 48 hours after sporozoite inoculations. Dot plots indicate the diameter of each liver stage parasite from two of the experiments shown in Fig. 3B. The horizontal lines show the median (long line) and interquartile range (short bars). At least 20 liver stage parasites were counted in each parasite line. The statistical difference was calculated by Kruskal-Wallis followed by Dunn's post-hoc test (*P < 0.05, ***P < 0.001, ns: no statistical difference).

Fig. S7 Number of gliding cycles.

Dot plots show the number of gliding cycles per sporozoite during five minutes of observation in five independent experiments. The long horizontal lines indicate the median number of gliding cycles and the short lines indicate the quartile range. Statistical differences were calculated by Kruskal-Wallis and Dunn post hoc tests (*$P < 0.05$, ***$P < 0.001$, ****$P < 0.0001$).

Fig. S8 RON4 were detected at the tip of sporozoites without permeabilization.

RON4-cont sporozoites were collected from salivary glands and to initiate gliding were incubated with RPMI 1640 medium containing 10% FCS for 1 hour in eight well chamber slides. The sporozoites were washed with PBS and fixed with 2% PFA followed by immunofluorescence staining with anti-RON4 (green) and anti-CSP (purple) antibodies. Nuclei are visualized with Hoechst 33342 in merged images (blue). Secreted RON4 was detected at the tip of sporozoites during gliding (asterisk). Bars, 5 μm.

Fig. S9 Antibodies against RON4 inhibited sporozoite infection of hepatocytes in a dose-dependent manner.

Thirty thousand WT sporozoites collected from salivary glands were inoculated onto HepG2 cells cultured in an eight-well chamber slide. The cells were incubated for 1 hour with 62.5 μg/ml, 125 μg/ml, 250 μg/ml, 500 μg/ml, or 1 mg/ml of anti-RON4 antibody or PBS in culture medium. Cells were washed with culture medium three times and then incubated for 5 hours with culture medium. After a total 6 hours of incubation, cells were washed with PBS and fixed with 10% formalin and permeabilized by 0.1% Triton X-100. The parasites were detected by IFA using antibodies against UIS4 and CSP. The mean values of PVM-positive parasites are shown as bars with deviation as error bars, from two wells for each antibody treatment. The experiment was repeated twice.
